# Temporal Changes in Effect Sizes of Studies Comparing Individuals With and Without Autism: A Meta-analysis

**DOI:** 10.1001/jamapsychiatry.2019.1956

**Published:** 2019-08-21

**Authors:** Eya-Mist Rødgaard, Kristian Jensen, Jean-Noël Vergnes, Isabelle Soulières, Laurent Mottron

**Affiliations:** 1 Department of Psychology, University of Copenhagen, Copenhagen, Denmark; 2 The Novo Nordisk Foundation Center for Biosustainability, Technical University of Denmark, Kgs Lyngby, Denmark; 3 Département de Prévention, Épidémiologie, Économie de la Santé, Odontologie Légale, Université Toulouse III-Paul-Sabatier, Faculté de Chirurgie Dentaire/CHU de Toulouse, Toulouse, France; 4 Division of Oral Health and Society, Faculty of Dentistry, McGill University, Montréal, Québec, Canada; 5 Département de Psychologie, Université du Québec à Montréal, Montréal, Québec, Canada; 6 Département de Psychiatrie, Université de Montréal, Montréal, Québec, Canada; 7 Centre de Recherche du CIUSSS-NIM, Hôpital Rivière-des-Prairies, Montréal, Québec, Canada

## Abstract

**Importance:**

The definition and nature of autism have been highly debated, as exemplified by several revisions of the *DSM* (*DSM-III, DSM-IIIR, DSM-IV*, and *DSM-*5) criteria. There has recently been a move from a categorical view toward a spectrum-based view. These changes have been accompanied by a steady increase in the prevalence of the condition. Changes in the definition of autism that may increase heterogeneity could affect the results of autism research; specifically, a broadening of the population with autism could result in decreasing effect sizes of group comparison studies.

**Objective:**

To examine the correlation between publication year and effect size of autism-control group comparisons across several domains of published autism neurocognitive research.

**Data Sources:**

This meta-analysis investigated 11 meta-analyses obtained through a systematic search of PubMed for meta-analyses published from January 1, 1966, through January 27, 2019, using the search string *autism* AND (*meta-analysis* OR *meta-analytic*). The last search was conducted on January 27, 2019.

**Study Selection:**

Meta-analyses were included if they tested the significance of group differences between individuals with autism and control individuals on a neurocognitive construct. Meta-analyses were only included if the tested group difference was significant and included data with a span of at least 15 years.

**Data Extraction and Synthesis:**

Data were extracted and analyzed according to the Preferred Reporting Items for Systematic Reviews and Meta-analyses (PRISMA) reporting guideline using fixed-effects models.

**Main Outcomes and Measures:**

Estimated slope of the correlation between publication year and effect size, controlling for differences in methods, sample size, and study quality.

**Results:**

The 11 meta-analyses included data from a total of 27 723 individuals. Demographic data such as sex and age were not available for the entire data set. Seven different psychological and neurologic constructs were analyzed based on data from these meta-analyses. Downward temporal trends for effect size were found for all constructs (slopes: –0.067 to –0.003), with the trend being significant in 5 of 7 cases: emotion recognition (slope: –0.028 [95% CI, –0.048 to –0.007]), theory of mind (–0.045 [95% CI, –0.066 to –0.024]), planning (–0.067 [95% CI, –0.125 to –0.009]), P3b amplitude (–0.048 [95% CI, –0.093 to –0.004]), and brain size (–0.047 [95% CI, –0.077 to –0.016]). In contrast, 3 analogous constructs in schizophrenia, a condition that is also heterogeneous but with no reported increase in prevalence, did not show a similar trend.

**Conclusions and Relevance:**

The findings suggest that differences between individuals with autism and those without the diagnosis have decreased over time and that possible changes in the definition of autism from a narrowly defined and homogenous population toward an inclusive and heterogeneous population may reduce our capacity to build mechanistic models of the condition.

## Key Points

**Question:**

Did effect sizes for group-level differences between individuals with autism and control individuals decrease during past decades?

**Findings:**

In this meta-analysis of 11 meta-analyses, effect sizes for 7 distinct differences between groups with autism and control groups decreased over time, with 5 of 7 being statistically significant.

**Meaning:**

The findings suggest that differences between individuals with autism and those without autism have decreased over time, which may be associated with changes in diagnostic practices.

## Teaser

This meta-analysis assesses effect sizes for statistically significant group-level differences between individuals with autism and control individuals for 5 distinct psychological constructs and 2 neurologic markers.

## Introduction

Autism was first described in the 1940s,^1^ and the definition of the condition has been the subject of much debate.^2^ The diagnostic criteria for autism have been revised several times, and our understanding of autism has evolved from a narrowly defined clinical picture to a spectrum of conditions of uncertain similarity. There has been an increase in the prevalence of autism from less than 0.05% in 1966^3^ to 1.47% among children aged 8 years in the United States^4^ and to more than 2% in studies^5^ measuring lifetime prevalence through less stringent case ascertainment. In the absence of a reliable biomarker for the diagnosis of autism, this statistic may reflect multiple factors, such as a true increase in autism in the population, greater public awareness of autism,^6^ diagnostic substitution,^7^ a link between diagnosis and support, greater tendency to diagnose individuals with an IQ in the normal range,^8^ a diminished threshold for clinical diagnosis,^9^ the use of checklist diagnoses,^10^ or low specificity of standardized diagnostic instruments in clinical settings.^11,12^ Possible changes in diagnostic practices may have resulted in empirical studies assessing an increasingly heterogeneous population, including individuals with less profound deviations from normal that would not have previously been classified as autistic.

We examined how this temporal change in the definition and clinical practices of autism might affect the ability of the scientific community to detect neurocognitive and neurologic differences between autistic and control samples. We predicted that the magnitude of group differences in studies comparing people with and without autism would depend on the period in which it was conducted and, more specifically, become smaller over time. We investigated whether a temporal decrease in effect size could be detected in a variety of cognitive neuroscience constructs commonly studied in autism and associated with group differences. We also studied the temporal evolution of similar variables in schizophrenia, a heterogeneous condition with stable prevalence, to differentiate temporal trends specific to autism from possible confounding factors.

## Methods

### Selection of Data Material

Meta-analyses of various neurocognitive constructs for which a group difference between those individuals with autism and comparison groups has been identified were used to investigate the correlation between effect size and publication year. The use of meta-analyses facilitated the identification of studies that tested the same or very similar group differences. Meta-analyses also tested the overall statistical significance of the given difference across studies, allowing constructs for which the difference is not significant to be excluded from the analysis because no temporal trend in effect size would be expected.

Potential meta-analyses were found through PubMed using the search string *autism* AND (*meta-analysis* OR *meta-analytic*). The search spanned the inception of the database (January 1, 1966) through January 27, 2019. The results were reduced to a set of candidate meta-analyses that were written in English, investigated group-level differences between individuals with autism and control groups, and included data on effect size, sample size, and relevant task or method for each primary study. The resulting meta-analyses were organized by the psychological constructs that were investigated (eg, theory of mind) and domain (eg, the social domain). Other inclusion criteria for the meta-analyses were a span of at least 15 years investigating a construct for which at least 1 meta-analysis found a statistically significant difference between a group with autism and a control group. Only domains for which at least 2 constructs could be analyzed were included to determine whether a temporal trend was systematically present or absent within a domain.

### Data Extraction

**Figure 1.**
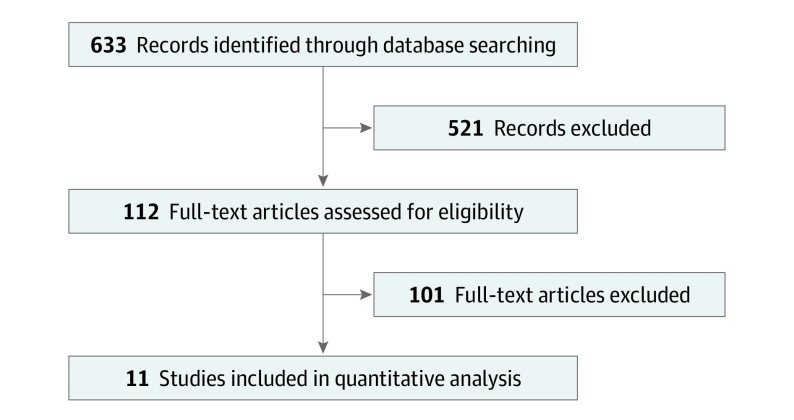
PRISMA Flowchart

The data selection process is outlined in Figure 1, and analyzed studies are listed in eTables 1-10 in the Supplement. Group difference effect sizes, sample sizes, and task or method for each study were obtained from the meta-analyses. Within each meta-analysis, primary studies were excluded if they used an invalid control group or an improper outcome metric or if other elements of the study design did not allow it to be meaningfully compared with the rest of the studies (eTable 11 in the Supplement). In addition, mean IQ and autistic group diagnosis (autism vs autism spectrum) (eTables 12-19 in the Supplement) were recorded for each primary study.

### Assessment of Data Quality

The overall quality of the literature searches of included meta-analyses was assessed according to criteria of the Cochrane Collaboration.^13,14^ These criteria make it possible to rate literature searches and the reproducibility of search strategies in meta-analyses. Publication bias was assessed using both original results from meta-analyses and funnel plots aggregating data for each construct. The quality of each primary study was rated using a tailored adaptation of the Newcastle-Ottawa Scale (eTable 19 in the Supplement).^15^

### Statistical Analysis

#### Quantification of the Temporal Effect Size Trend

A multivariable linear regression model, which is a sensitive method for detecting gradual changes in effect sizes,^16^ was fitted with effect size as the dependent variable. Publication year was included as an independent variable along with the task or method used (eg, strange stories) because different task variants could be expected to give systematically different effect sizes. Although the expected effect size should in theory be invariant to changes in sample size,^17^ publication bias might cause small studies to systematically report larger effect sizes than large studies.^18^ Sample size was also included in the regression analysis to control for such bias. The estimated slope of the correlation between publication year and effect size was used as the outcome to quantify the temporal effect size trend, and *F* tests were used to quantify the statistical significance of the temporal trend in each individual construct. Furthermore, the association of Newcastle-Ottawa Scale quality score, group comparability score, IQ difference, and autism-group diagnosis with the magnitude of effect sizes was tested by individually adding them to the model and performing *F* tests. All statistical analyses were conducted in Python 3.5 (Python Software Foundation) using the statsmodels package. Statistical tests were performed as 2-tailed tests with a significance level of .05.

#### Proteus Phenomenon

Each construct was examined for the presence of the Proteus phenomenon,^19^ a situation in which the first reported effect size in a given area of study is unrealistically large because of publication bias. This publication bias leads to the earliest effect size being larger than that which can be explained by the regression model. The presence of the Proteus phenomenon was tested by calculating the studentized residuals of the first study for each task. A studentized residual with a *t* value above the 95th percentile was considered to be evidence of the Proteus effect. This is an adaptation of the method described by Koricheva et al,^16^ which works in the presence of moderator variables.

#### Nonautistic Comparison Group

Observed temporal trends could be specific to autism or representative of a general trend across diagnostic categories. A control analysis was performed using data comparing individuals with schizophrenia with the typical population. Schizophrenia was chosen because some neurocognitive deficits, such as theory of mind and executive functioning, have been identified in both groups.^20,21^ However, the prevalence of schizophrenia has remained stable for the past 2 decades.^22^ Meta-analyses for schizophrenia were selected to match those selected for autism as closely as possible.

## Results

We found 11 meta-analyses^20,23,24,25,26,27,28,29,30,31,32^ comprising 7 constructs within 3 domains: social (emotion recognition and theory of mind), executive (cognitive flexibility, planning, and inhibition), and neurologic (event-related potential P3b and brain size) (Table). These included a total of 27 723 individuals.

**Table.** Overview of the Results for the 7 Constructs in Autism and the 3 Constructs in Schizophrenia

Construct	Source	*R*^2^	Year, Slope^a^	*P* Value^b^	
				Year	Participants
Autism					
Social domain^c^					
Emotion recognition	Chung et al,^20^ 2014	0.28	−0.028	.005	.27
	Leppanen et al,^23^ 2018				
	Peñuelas-Calvo et al,^24^ 2019				
	Uljarevic and Hamilton,^25^ 2013				
Theory of mind	Chung et al,^20^ 2014	0.54	−0.045	<.001	.16
	Leppanen et al,^23^ 2018				
Executive domain^c^					
Planning	Olde Dubbelink and Geurts,^26^ 2017	0.54	−0.067	.03	.45
	Lai et al,^27^ 2017				
Cognitive flexibility	Landry and Al-Taie,^28^ 2016	0.11	−0.013	.18	.45
	Lai et al,^27^ 2017				
	Westwood et al,^29^ 2016				
Inhibition	Geurts et al,^30^ 2014	0.07	−0.003	.82	.97
	Lai et al,^27^ 2017				
Neurologic domain^c^					
P3b amplitude	Cui et al,^31^ 2017	0.65	−0.048	.02	.83
Brain size	Sacco et al,^32^ 2015	0.41	−0.047	.003	.77
Schizophrenia					
Theory of mind	Chung et al,^20^ 2014	0.35	−0.008	.37	.06
	Bora et al,^33^ 2009				
Inhibition, Stroop task	Westerhausen et al,^34^ 2011	0.23	0.011	.23	.89
Gray matter volume	Haijma et al,^35^ 2013	0.02	0.008	.42	.39

^a^ Slope denotes the regression coefficient for the year variable in the linear models with effect size as the outcome variable.

^b^ *P* values denote the significance of the association of the year variable and participants with effect sizes and are calculated using *F* tests on the linear models with effect size as the outcome variable.

^c^ Some meta-analyses included more than 1 construct.

### Quality of the Data

Selection criteria of the primary studies in the 11 meta-analyses are reported in eTables 21-24 in the Supplement, with good comparability among meta-analyses. Inclusion periods largely overlapped (publication years of meta-analyses between 2013 and 2018), indicating a low risk of bias because of the heterogeneity of data sources (eTables 21-23 and 25 in the Supplement). The mean score of the quality of the literature search strategies of the meta-analyses was 5.5 (range, 3.0-8.0) on the 9-item scale, in which higher numbers are considered to indicate better quality (eTable 26 in the Supplement). There was some evidence of publication bias for the 2 constructs of the social domain but not the other constructs (eTable 27 and eFigure in the Supplement).

### Autism

**Figure 2.**
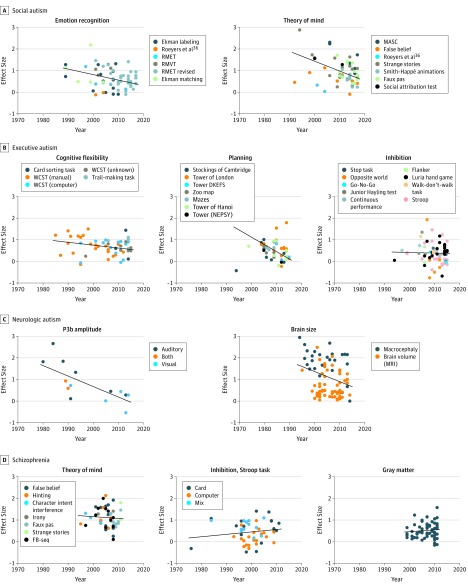
Overview of the Development of Effect Sizes Over Time Within Each of the Constructs Each point represents an effect size originating from an empirical study. Colors indicate which task or method was used within the study. The black line indicates the fitted linear model. In the analysis of planning and inhibition, method type was defined as the combination of task and outcome metric. Points are colored by task alone for the purpose of visualization. DKEFS indicates Delis-Kaplan Executive Function System; FB-seq, False Belief sequencing task; MASC, Movie for Assessment of Social Cognition; MRI, magnetic resonance imaging; NEPSY, Developmental Neuropsychological Assessment; RMET, Reading the Mind in the Eyes Test; RMVT, Reading the Mind in the Voice Test; and WCST, Wisconsin Card Sorting Task.

**Figure 3.**
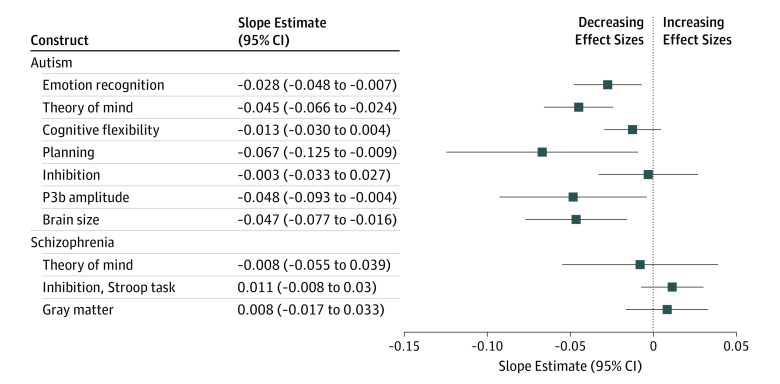
Forest Plot of the Estimated Change in Effect Size per Year

The results of the statistical analysis of the 7 neurocognitive constructs are shown in the Table, and the correlations between publication year and effect size are shown in Figure 2^36^ (see eResults in the Supplement for a detailed description of the results for each construct). The slope estimates for publication year were negative for all 7 constructs (Figure 3), indicative of a general tendency for the effect size to decrease over time. The regression models showed that the association of publication year with effect size was statistically significant for 5 of 7 constructs: emotion recognition (slope: –0.028 [95% CI, –0.048 to –0.007]), theory of mind (–0.045 [95% CI, –0.066 to –0.024]), planning (–0.067 [95% CI, –0.125 to –0.009]), P3b amplitude (–0.048 [95% CI, –0.093 to –0.004]), and brain size (–0.047 [95% CI, –0.077 to –0.016]). For the cognitive flexibility construct, effect sizes from 1 primary study^37^ deviated substantially from those of almost all other studies. This unusual result was also noted by the author, and a reproduction of the study^37^ did not replicate it, instead reporting findings consistent with the remaining literature. If the abnormal effect sizes were excluded from our analysis, the results changed markedly, with the association of publication year with effect size also becoming significant for this construct (slope, –0.018; *P* = .02).

There was evidence of the Proteus phenomenon for only 1 of the included tasks (the strange stories task within the theory of mind construct), but when the analysis was rerun without the outlying effect size,^38^ the decrease in effect size over time was still significant and prominent (*P* < .001) (see eResults in the Supplement). This suggests that the decreasing trends in effect size were not merely explained by the first studies overestimating the effect size. Instead, there appeared to be a steady decrease throughout the examined period.

### Schizophrenia

We performed a similar analysis on 4 meta-analyses investigating group-level differences between individuals with schizophrenia and controls. The constructs investigated were theory of mind, cognitive inhibition (Stroop task), and gray matter volume, abnormalities in all of which were found by the meta-analyses to be significantly associated with schizophrenia. The data for the theory of mind analysis were obtained from meta-analyses conducted by Chung et al^20^ and Bora et al.^33^ Data to explore the constructs of cognitive inhibition and gray matter volume were extracted from meta-studies conducted by Westerhausen et al^34^ and Haijma et al,^35^ respectively. The results of the analysis are shown in the Table, and correlation plots for publication year and group-level effect size for the 3 constructs are shown in Figure 2. The temporal trends were not significant for any of the constructs.

### Study Quality, Other Variables, and the Temporal Trend of Effect Size

We tested whether the temporal trend in effect size could be explained by systematic changes in study design over time by testing the association of quality score, group comparability score, group IQ difference, and autism diagnosis type with effect size. There was no significant association with group difference effect size for quality score or comparability score as measured by an adapted Newcastle-Ottawa Scale (eTable 19 in the Supplement), and control for these variables did not alter the significance of the correlation with publication year (eTable 20 in the Supplement). Group IQ differences were significantly associated with effect size only for the 2 constructs for which no temporal trend was identified initially (cognitive flexibility and inhibition). Among the remaining constructs, IQ differences did not have a significant correlation with effect size and the significance of the associations between publication year and effect size was not altered.

Testing how differences in the definition of autism diagnosis were associated with group differences proved to be difficult because different authors used different systems of classification for individuals with autism (eg, autism, high-functioning autism, and Asperger syndrome). Older studies mostly included individuals with an “autism” diagnosis, whereas newer studies more often used mixtures of people with an autism, Asperger, or an “autism spectrum disorder” diagnosis. Whether a study used or did not use a sample with pure autism (or high-functioning autism) was not, however, significantly associated with group difference effect sizes for any of the constructs, and including this variable in the analysis did not change the significance of the association between publication year and effect size.

## Discussion

We investigated effect sizes for 5 distinct psychological constructs and 2 neurologic markers for which statistically significant group-level differences between individuals with autism and control individuals have previously been identified. We found that effect sizes decreased over the past 2 decades. The relative decrease in mean effect size from 2000 through 2015 ranged from 45% to more than 80% among the constructs for which the temporal decrease was significant. The trend observed for autism deviated from that observed for schizophrenia, another psychiatric condition with comparable absolute prevalence but for which there was no documented increase in prevalence during the investigated period.

Changes in our understanding and the definition of autism may have occurred in different ways. One factor could be the evolution of diagnostic criteria associated with a gradual expansion in our understanding and the definition of autism. This may have introduced additional extrinsic heterogeneity. As an example, attention-deficit/hyperactivity disorder was considered to be a differential diagnosis in the *DSM-IV*, whereas it is listed as a possible co-occurring condition in the *DSM-5*, so that the social effect of severe attention-deficit/hyperactivity disorder may be mistaken for autism.^39^ Increased attention-deficit/hyperactivity disorder comorbidity could explain why the temporal decrease appeared to be smaller for executive compared with social or neurologic constructs. However, although executive deficits are shared by the 2 conditions, they may encompass distinct executive functions^40^ and imperfectly overlap with clinical traits.^41^

Given that the decrease in effect size appeared to be gradual rather than stepwise and occurred largely within the *DSM-IV* criteria era (1994-2013) (Figure 2), changes in diagnostic criteria alone cannot explain such a trend. Another factor might be that individuals with autism included in research are becoming decreasingly distinct from typical comparison groups. For example, the threshold for recognizing each individual criterion may have been lowered, such that a lesser degree of each autistic symptom is necessary for a diagnosis of autism. In support of this interpretation, 1 study found that children in Sweden aged 7 to 12 years who received a diagnosis of autism in 2014 had a 50% lower autism symptom score than did those diagnosed in 2004, whereas the prevalence of autism simultaneously increased 5-fold in this age group.^9^ In parallel, the pool of individuals with autism diagnoses from which participants in research experiments are extracted generally satisfy Autism Diagnostic Interview and Autism Diagnostic Observation Schedule criteria, a set of criteria for which there can be problems with reliability^10,42^ and specificity.^43,44^ Regardless of the cause, the hypothesis of a broadening understanding of autism is consistent with the marked increase in the prevalence of autism that has been observed in recent decades and attributed, among other possibilities, to less stringent case ascertainment.

Another potential reason for decreasing effect sizes is changes in study design quality over time, such that older studies may not have controlled for age or IQ as strictly as newer studies. Our results from rating the quality of the primary studies suggest that the observed decrease in effect size cannot be explained by changes in study design, measured as either full quality score, specific group comparability score, group IQ difference, or diagnostic type.

The finding of group-level differences in specific constructs has led to the development of intervention practices that target such differences, such as theory of mind. However, a meta-analysis of interventions focused on improving theory of mind in autism showed a lack of efficacy.^45^ More generally, systematic reports on intervention effects do not argue for an effect across all psychological constructs investigated in this study.^46^

In our analysis of effect size, we stratified the studies based on the task that was used to measure the construct under study. This ensured that only studies using the same methods were compared. However, authors sometimes make minor alterations to task procedures to explore specific research questions, which may affect the observed group difference. This would most likely result in a random change in effect size rather than the consistent decrease that we observed here. However, if the methodologic changes are applied systematically, over time, they may become confounded with the association of publication year. The risk of confounding associations of small alterations of specific tests is difficult to completely eliminate, because a strict grouping of studies based on the use of the same procedure would probably leave few studies within each group, thus precluding analysis of temporal trends within each group.

The phenomenon of changes in effect sizes over time has been investigated outside the autism domain by Ioannidis and Trikalinos.^19^ An observed decrease over time could theoretically be associated with the Proteus phenomenon, as described in the Methods section. Our analysis shows that pioneering studies did not generally find abnormally large effect sizes compared with the studies that followed. Only the strange stories task showed evidence of the Proteus phenomenon, but exclusion of the study in question^38^ did not change the results markedly.

Monsarrat and Vergnes^47^ have explored the general evolution of published effect sizes within the biomedical sciences and found a consistent and significant decreasing trend in effect size. This finding may be associated with the increasing pressure to publish seen in all fields of research during the past decades. Although this trend is also likely to apply to autism research, our results showed temporal decreases in effect size, with mean slopes being an order of magnitude larger than the global decrease observed by Monsarrat and Vergnes.^47^ This result suggests the presence of some mechanism specific to the field of autism.

After a period in which cognitive models of autism were prevalent, the inability to consistently replicate previous findings resulted in meta-analyses being conducted to test the robustness of the early findings on which the models were based. In general, such meta-analyses have found the associations to be more modest than those originally reported, casting doubt on the generality of the previously reported cognitive deficits. This has been the case for executive function deficits^48^ as well as cognitive correlates of social functioning, including theory of mind deficits^49^ and visuo-spatial peaks of performance.^50^ Before claiming that a finding obtained in the previous decades was simply a type 1 error or inflated because of publication bias, the possibility of a temporal decrease in effect size should be considered. Thus, it is possible that the heterogeneity in the autistic population used in research has detrimental consequences for the understanding of autistic neurocognitive mechanisms. The use of an inclusive autism spectrum disorder category for research participants could result in a diffused mean that masks potentially diverse mechanisms observable only through the use of more homogenous subgroups.

It may be insufficient to match groups on the variables commonly used for this purpose (age, sex, and intelligence) to reduce the noise introduced by the heterogeneity of the groups under study. Although the study of individuals representing many different expressions of autism, including those with milder autistic presentations, is justified and useful, future research in cognitive neuroscience could benefit from focusing on the identification of meaningful subtypes within the autism spectrum. Progress toward this could be achieved by studying the structure of the correlation between different traits and characteristics associated with autism to find phenotypic clusters. Meaningful subgroups of autism could potentially be identified by the presence or absence of speech onset delay,^51^ neurogenetic conditions,^52^ or nonverbal intellectual disability.^53^ Clinical specifiers, such as those described in the *DSM-5* autism spectrum disorder diagnosis, could therefore be used to stratify autism into more homogenous subgroups rather than as a mere description of an accepted heterogeneity of the autism spectrum. Potential subgroups could be studied separately and jointly, representing a categorical and a dimensional approach^54^ of autistic heterogeneity.

Pioneers of a spectrum view of autism argued that research on narrowly defined subtypes is of limited value because findings can only be generalized to a small group of individuals.^55^ However, gradual changes to a diagnostic category, such as autism, and blurring of the distinction between autistic traits and autism^56^ could potentially affect our ability to advance mechanistic models of the condition. The belief within autism research that large heterogeneous populations are preferable compared with small narrowly defined ones in the search for scientific truth may be open to question.

### Limitations

The constructs studied did not cover the entire range of domains for which autistic differences have been found in cognitive neuroscience. In particular, they did not encompass affective neuroscience, language, or repetitive behaviors. This leaves open the possibility that differences in these domains may be more stable in terms of the mechanisms responsible for the observed decrease in effect size. Future studies may need to use complementary methods to broaden the coverage of autistic features because group-level comparisons within these domains are not as numerous as for the domains included here.

## Conclusions

The findings suggest that differences between individuals with autism and controls have decreased over time, which might be associated with changes in the definition of autism from a narrowly defined population toward an inclusive and heterogeneous population. This could have implications for our ability to build mechanistic models of the autism condition.

## Notes

Supplement. eTable 1. Data for Emotion Recognition (Autism) eTable 2. Data for Theory of Mind (Autism) eTable 3. Data for Cognitive Flexibility (Autism) eTable 4. Data for Planning (Autism) eTable 5. Data for Inhibition (Autism) eTable 6. Data for P3b Amplitude (Autism) eTable 7. Data for Brain Size (Autism) eTable 8. Data for Theory of Mind (Schizophrenia) eTable 9. Data for Stroop Task (Schizophrenia) eTable 10. Data for Grey Matter Volume (Schizophrenia) eTable 11. List of Excluded Primary Studies eTable 12. NOS Ratings for Emotion Recognition eTable 13. NOS Ratings Theory of Mind eTable 14. NOS Ratings for Cognitive Flexibility eTable 15. NOS Ratings for Planning eTable 16. NOS Ratings for Inhibition eTable 17. NOS Ratings for P3b Amplitude eTable 18. NOS Ratings for Brain Size eTable 19. NOS Rating Criteria eTable 20. Results of Analysis of Control Variables eTable 21. Quality of Meta-analyses—Social Domain eTable 22. Quality of Meta-analyses—Executive Domain eTable 23. Quality of Meta-analyses—Neurological Domain eTable 24. Comparison of Meta-analysis Quality eTable 25. Meta-analyses: Databases and Search Strategies eTable 26. Reproducibility and Quality of the Search Strategies in Meta-analyses eTable 27. Assessment of Publication Bias in Meta-analyses eFigure. Aggregate Publication Bias per Construct eResults. Supplementary Results
