# Supplementary material for: Temporal Changes in Effect Sizes of Studies Comparing Individuals With and Without Autism: A Meta-analysis
Source: JAMA Psychiatry. 2019 Aug 21;76(11):1124–32. doi: 10.1001/jamapsychiatry.2019.1956 (PMC6704749; doi:10.1001/jamapsychiatry.2019.1956)
Supplement: Supplement. — eTable 1. Data for Emotion Recognition (Autism) eTable 2. Data for Theory of Mind (Autism) eTable 3. Data for Cognitive Flexibility (Autism) eTable 4. Data for Planning (Autism) eTable 5. Data for Inhibition (Autism) eTable 6. Data for P3b Amplitude (Autism) eTable 7. Data for Brain Size (Autism) eTable 8. Data for Theory of Mind (Schizophrenia) eTable 9. Data for Stroop Task (Schizophrenia) eTable 10. Data for Grey Matter Volume (Schizophrenia) eTable 11. List of Excluded Primary Studies eTable 12. NOS Ratings for Emotion Recognition eTable 13. NOS Ratings Theory of Mind eTable 14. NOS Ratings for Cognitive Flexibility eTable 15. NOS Ratings for Planning eTable 16. NOS Ratings for Inhibition eTable 17. NOS Ratings for P3b Amplitude eTable 18. NOS Ratings for Brain Size eTable 19. NOS Rating Criteria eTable 20. Results of Analysis of Control Variables eTable 21. Quality of Meta-analyses—Social Domain eTable 22. Quality of Meta-analyses—Executive Domain eTable 23. Quality of Meta-analyses—Neurological Domain eTable 24. Comparison of Meta-analysis Quality eTable 25. Meta-analyses: Databases and Search Strategies eTable 26. Reproducibility and Quality of the Search Strategies in Meta-analyses eTable 27. Assessment of Publication Bias in Meta-analyses eFigure. Aggregate Publication Bias per Construct eResults. Supplementary Results [file jamapsychiatry-76-1124-s001.pdf]

Supplementary Online Content

Rødgaard E-M, Jensen K, Vergnes J-N, Soulières I, Mottron L. Temporal changes in effect sizes of studies comparing individuals with and without autism: a meta-analysis. *JAMA Psychiatry*. Published online August 21, 2019.
doi:10.1001/jamapsychiatry.2019.1956

eTable 1. Data for Emotion Recognition (Autism)

eTable 2. Data for Theory of Mind (Autism)

eTable 3. Data for Cognitive Flexibility (Autism)

eTable 4. Data for Planning (Autism)

eTable 5. Data for Inhibition (Autism)

eTable 6. Data for P3b Amplitude (Autism)

eTable 7. Data for Brain Size (Autism)

eTable 8. Data for Theory of Mind (Schizophrenia)

eTable 9. Data for Stroop Task (Schizophrenia)

eTable 10. Data for Grey Matter Volume (Schizophrenia)

eTable 11. List of Excluded Primary Studies

eTable 12. NOS Ratings for Emotion Recognition

eTable 13. NOS Ratings Theory of Mind

eTable 14. NOS Ratings for Cognitive Flexibility

eTable 15. NOS Ratings for Planning

eTable 16. NOS Ratings for Inhibition

eTable 17. NOS Ratings for P3b Amplitude

eTable 18. NOS Ratings for Brain Size

eTable 19. NOS Rating Criteria

eTable 20. Results of Analysis of Control Variables

eTable 21. Quality of Meta-analyses—Social Domain

eTable 22. Quality of Meta-analyses—Executive Domain

eTable 23. Quality of Meta-analyses—Neurological Domain

eTable 24. Comparison of Meta-analysis Quality

eTable 25. Meta-analyses: Databases and Search Strategies

eTable 26. Reproducibility and Quality of the Search Strategies in Meta-analyses

eTable 27. Assessment of Publication Bias in Meta-analyses

eFigure. Aggregate Publication Bias per Construct

eResults. Supplementary Results

© 2019 Rødgaard E-M et al. *JAMA Psychiatry.*

This supplementary material has been provided by the authors to give readers additional information about their work.

© 2019 Rødgaard E-M et al. *JAMA Psychiatry.*

eTable 1. Data for Emotion Recognition (Autism)

The meta-analysis by Chung et al. only included a coarse annotation of the specific method used in each study. Studies identified from this meta-analysis only were examined and annotated with a task in accordance with the task categories obtained from the remaining three meta-analyses of the emotion recognition construct.

From the meta-analysis by Uljarevic & Hamilton, only studies using the Ekman task were included, as the remaining studies used a wide range of test procedures. Both of the Ekman variants (Emotion Labelling and Emotion Matching) were included.

Title	Year	Type	Effect	Participants
Adler et al.	2010	RMET revised	0.7	37
Baron-Cohen	2015	RMET revised	0.5	715
Baron-Cohen et al.	2001	RMET revised	1.69	29
Baron-Cohen et al.	1997	Old RMET	1.415	32
Baron-Cohen et al.	1999	Old RMET	1.467	20
Bolte & Poustka	2003	Ekman EL	1.0538	37
Boraston	2007	Ekman EL	0.5923	20
Braverman	1989	Ekman EM	1.3403	20
Braverman	1989	Ekman EL	0.9536	20
Brent et al.	2004	RMET revised	0.67	40
Buitelaar	1999	Ekman EM	0.6976	40
Castelli	2005	Ekman EL	0.2177	40
Celani	1999	Ekman EM	2.4172	20
Clark	2008	Ekman EL	1.2005	25
Corden	2008	Ekman EL	0.4175	42
Couture et al.	2010	RMET revised	0.61	77
Craig et al.	2004	RMET revised	1.43	33
David et al.	2008	RMET revised	1.23	48
Demurie et al	2011	RMET revised	0.9	31
Dziobek	2006	Ekman EL	1.5134	34
Dziobek et al	2006	RMET revised	1.5	41
Golan et al.	2007	RMVT	1.34	72
Golan et al.	2007	RMET revised	1.1	72
Gonzalez-Gadea et al	2013	RMET revised	0.13	44
Grossman	2000	Ekman EL	0.2898	26
Holt et al.	2014	RMET revised	0.72	89
Jones	2010	Ekman EL	0.1367	152

Kaland et al.	2008	RMET revised	0.85	41
Kirchner et al.	2011	RMET revised	0.73	41
Kleinman et al.	2001	RMVT	1.29	31
Kleinman et al.	2001	Old RMET	0.61	48
Kritsten et al	2014	RMET revised	0.65	40
Lahera et al	2014	RMET revised	1.64	48
Lai	2012	RMET revised	1	128
Lehnhardt et al.	2011	RMET revised	0.98	78
Lombardo	2007	RMET revised	0.6	60
Lugnegard et al	2013	RMET revised	0.28	103
Macdonald	1989	Ekman EL	1.5052	20
Muller	2016	RMET revised	0.37	56
Murray et al	2017	RMET revised	0.63	40
Otsuka et al.	2017	RMET revised	1	42
Pedreno	2017	RMET revised	0.62	70
Pelphrey	2002	Ekman EL	1.5042	10
Peterson et al.	2015	RMET revised	0.64	75
Phillip	2009	Ekman EL	1.5478	46
Piggot	2004	Ekman EL	0.7435	28
Piggot	2004	Ekman EM	0.7038	28
Ponnet et al. rewritten SS	2004	Alternative	0.21	38
Robinson et al	2017	RMET revised	0.64	48
Roeyers et al. Rewritten SS	2001	Alternative	0.11	48
Rosenblau et al	2015	RMET revised	0.72	51
Rueda et al.	2015	RMET revised	0.5	76
Rutherford	2002	RMVT	1.02	39
Sato	2017	RMET revised	0.89	38
Schaller & Rauh	2017	RMET revised	1.17	46
Schuwerk et al	2015	RMET revised	1.18	37
Segura	2015	RMET revised	0.53	31

© 2019 Rødgaard E-M et al. *JAMA Psychiatry.*

Spek et al.	2010	RMET revised	0.32	93
Tantam	1989	Ekman EL	0.9575	20
Torralva et al	2013	Old RMET	0.37	61
Vogindroukas	2014	RMET revised	0.84	80
Wallace, Coleman & Bailey expt 1	2008	Ekman EL	0.799	52
Wilson	2014	RMET revised	0.98	178
Wright	2008	Ekman EL	0.1403	70

© 2019 Rødgaard E-M et al. *JAMA Psychiatry.*

eTable 2. Data for Theory of Mind (Autism)

Title	Year	Type	Effect	Participants
Adler et al.	2010	Strange stories	0.69	37
Beversdorf	1998	FBT	0.91	23
Bowler	1992	FBT	0.46	30
Brewer	2017	FPT	0.6	243
Brewer et al.	2017	Strange stories	0.73	243
Brewer et al.	2017	SHA	0.24	243
Brown and Klein	2011	SAT	1.28	32
Brunsdon	2015	FBT	0.52	341
Brunsdon et al.	2015	SHA	0.24	341
Crane	2013	Strange stories	0.58	56
David et al.	2008	Strange stories	1.58	48
Dziobek	2006	MASC	2.24	41
Dziobek	2006	Strange stories	0.77	41
Dziobek	2006	MASC	2.31	34
Flood	2011	Strange stories	0.7	50
Gonzalez-Gadea	2013	FPT	1.34	44
Grainger et al.	2014	SHA	0.83	36
Happe	1994	Strange stories	2.88	28
Heavey et al.	2000	Strange stories	1.6	31
Hill et al.	2004	FBT	1.13	30
Jolliffe & Baron-Cohen	1999	Strange stories	1.73	51
Klin	2000	SAT	1.56	60
Kristen	2014	Strange stories	1.21	40
Lahera	2014	MASC	1.34	48
Lahera	2014	Strange stories	1.63	48
Lever & Geurts	2016	FPT	0.41	236
Lind et al.	2014	SHA	0.62	56
Lugnegard et al.	2013	SHA	0.57	103
Marsh et al.	2011	SHA	0.76	37
Martinez	2017	MASC	1.73	39
Muller	2016	MASC	0.87	56
Murray	2017	Strange stories	0.61	40
Murray et al.	2017	SHA	0.79	40
Oakley	2016	MASC	1.1	43

© 2019 Rødgaard E-M et al. *JAMA Psychiatry.*

Pedreno	2017	FPT	0.78	70
Pedreno	2017	Strange stories	1.1	70
Ponnet et al.	2004	Alternative	0.04	38
Roeyers et al.	2001	Alternative	0.33	48
Scaller & Rauh	2017	FBT	0.41	45
Schaller & Rauh	2017	MASC	0.78	45
Schneider	2013	Strange stories	0.61	34
Schuwerk	2015	Strange stories	0.29	37
Segura	2015	Strange stories	1.07	31
Segura	2015	FPT	0.41	31
Senju	2009	Strange stories	0.25	36
Senju	2009	FBT	0.52	36
Spek	2010	FPT	0.76	93
Spek et al.	2010	Strange stories	0.81	93
Torralva	2013	FPT	1.44	61
White	2011	FBT	0.81	31
White	2014	FBT	0.83	33
White	2011	Strange stories	1.16	31
White et al.	2011	SHA	1.06	31
Wilson	2014	FBT	-0.09	178
Wilson et al.	2014	SHA	0.72	178
Yeh	2010	FPT	0.96	67
Yeh	2010	FBT	0.75	67
Yeh	2010	Strange stories	0.76	67
Zalla	2016	FPT	0.96	42
Zalla	2015	FPT	1.36	38
Zalla	2009	FPT	1.71	30
Zalla and Leboyer	2011	FPT	0.69	48

© 2019 Rødgaard E-M et al. *JAMA Psychiatry.*

eTable 3. Data for Cognitive Flexibility (Autism)

For the Wisconsin Card Sorting Task, only effect sizes based on perseverative errors were used.

Title	Year	Type	Effect	Participants
Ambery et al.	2006	WCST (Manual)	0.54	47
Chan et al. (a)	2011	WCST (Unknown)	0.71	40
Czermainski	2014	Trail Making Task	1.11	30
Geurts	2004	WCST (Computer)	0.86	80
Goddard	2014	WCST (Unknown)	0.49	126
Goldstein et al.	2001	WCST (Manual)	0.59	206
Greibling et al.	2010	WCST (Manual)	0.79	62
Hill & Bird	2006	WCST (Manual)	0.08	44
Kado et al.	2012	WCST (Manual)	0.43	104
Kaland et al.	2008	WCST (Manual)	0.24	26
Kilincaslan	2010	WCST (Computer)	0.89	30
Lam & Yeung	2012	WCST (Computer)	0.06	24
Li	2014	WCST (Computer)	0.58	69
Liss et al.	2001	WCST (Manual)	0.76	109
Lopez et al.	2005	WCST (Manual)	0.71	34
Maes et al	2011	WCST (Computer)	-0.044	36
Mean of Willam & Jarrold and Williams et al. (2013)	2013	WCST (Computer)	0.8	42
Minshew et al.	2002	WCST (Manual)	0.69	197
Minshew et al.	1997	WCST (Unknown)	0.24	66
Minshew et al.	1992	WCST (Manual)	0.38	30
Narzisi	2013	Card Sorting Task	1.44	66
Ozonoff & Jensen	1999	WCST	0.8	69

© 2019 Rødgaard E-M et al. *JAMA Psychiatry.*

		(Manual)		
Ozonoff & McEvoy	1994	WCST (Manual)	1.17	34
Ozonoff et al.	1991	WCST (Manual)	1.16	43
Ozonoff et al. study 2	1995	WCST (Manual)	-0.14	21
Ozonoff study 2	1995	WCST (Computer)	-0.07	21
Ozonoff study 3	1995	WCST (Computer)	0.84	24
Ozonoff study 3	1995	WCST (Manual)	1.11	24
Pascualvaca et al.	1998	WCST (Manual)	1.5	46
Perez	2009	Card Sorting Task	0.6	31
Prior	1990	WCST (Manual)	0.84	24
Robinson	2009	WCST (Computer)	0.38	108
Rumsey	1985	WCST (Manual)	1.21	19
Russel-smith	2014	Card Sorting Task	0.11	35
Sawa	2013	WCST (Unknown)	0.91	38
Schneider & Asarnow test 1	1987	WCST (Manual)	0.28	43
Semrud-Clikeman	2014	Card Sorting Task	0.58	74
Semrud-Clikeman	2014	Trail Making Task	1.02	74
Shu	2001	WCST (Computer)	0.94	78
Sumiyoshi	2011	WCST (Computer)	0.92	37
Szatmari et al.	1990	WCST (Manual)	1.41	79
Tsuchiya	2005	WCST (Computer)	0.99	42
Vanegas	2015	WCST (Unknown)	0.49	49
Verte	2006	WCST (Unknown)	0.81	159
Voelbel	2006	Trail Making Task	0.96	51
Voelbel	2006	WCST (Manual)	0.45	51
Winsler	2007	WCST	0.64	61

© 2019 Rødgaard E-M et al. *JAMA Psychiatry.*

		(Computer)		
Yang et al.	2009	WCST (Manual)	0.3	50
Yasuda et al.	2014	WCST (Computer)	0.9	66
van Eylen	2015	WCST (Unknown)	0.63	100
van Eylen	2011	WCST (Unknown)	0.52	80

© 2019 Rødgaard E-M et al. *JAMA Psychiatry.*

eTable 4. Data for Planning (Autism)

Title	Year	Type	Outcome	Effect	Participants
B_lte	2011	Tower of Hanoi	Total moves	-0.19	114
Boucher	2005	Zoo Map	Total score	0.76	20
Bramham	2009	Zoo Map	Accuracy	0.19	76
Corbett	2009	Stockings of Cambridge	Total perfect solutions	0.91	36
Geurts	2004	Tower of London	ToL score	0.78	82
Geurts & Vissers	2012	Tower of London	Excess moves	-0.23	46
Goddard	2014	Tower of London	Correct responses	0.49	126
Goldberg	2005	Stockings of Cambridge	Total perfect solutions	0.56	49
Griebling	2010	Tower of Hanoi	Total moves	0.95	75
Hanson & Atance	2014	Tower of Hanoi	Highest level achieved	0.09	50
Happe	2006	Stockings of Cambridge	Total perfect solutions	0.19	64
Hill & Bird	2006	Zoo Map	Accuracy	0.39	44
Hughes	1994	Stockings of Cambridge	Decision time	-0.43	74
Joseph	2005	Tower nepsy	Total perfect solutions	0.51	68
Kaufmann	2013	Stockings of Cambridge	Total perfect solutions	-0.04	20
Keary	2009	Tower of Hanoi	Total moves	0.93	66
Kimhi	2014	Tower of London	Total perfect solutions	0.58	59
Landa & Goldberg	2005	Stockings of Cambridge	Total perfect solutions	1.01	38
Limoges	2013	Tower of London	Total perfect solutions	0.64	31
Losh	2009	Tower of Hanoi	Total moves	0.27	77
Low	2009	Mazes	Accuracy	0.63	54
McCrimmon	2012	Tower DKEFS	Total score	0.07	66
Medeiros & Winsler	2014	Tower of Hanoi	Total moves	0.51	53
Ozonoff	2004	Stockings of Cambridge	Total perfect solutions	0.87	149
Ozonoff & Jensen	1999	Tower of Hanoi	Total score	0.7	69
Panerai	2014	Tower of London	Total perfect solutions	1.79	20
Pellicano	2010	Tower of London	Total perfect solutions	1.54	89
Pellicano	2007	Mazes	Accuracy	0.54	70
Pellicano	2006	Mazes	Accuracy	0.63	80

© 2019 Rødgaard E-M et al. *JAMA Psychiatry.*

Planche & Lemonnier	2012	Tower nepsy	Total score	-0.04	45
Rajendran	2005	Zoo Map	Summary profile score	0.68	24
Robinson	2009	Tower of London	Total moves	0.52	108
Sachse	2013	Stockings of Cambridge	Total perfect solutions	0.37	58
Schurink	2012	Tower of London	ToL score	0.6	56
Semrud-Clikeman	2010	Tower DKEFS	Total achievement	0.82	46
Sinzig	2008	Stockings of Cambridge	Total perfect solutions	0.07	40
Unterrainer	2015	Tower of London	Total perfect solutions	0.13	60
Van Eylen	2015	Tower DKEFS	Total score	0.2	100
Verte	2006	Tower of London	ToL score	0.68	159
Verte	2005	Tower of London	ToL score	0.82	108
Wallace	2009	Tower of London	Excess moves	0.63	53
White	2009	Zoo Map	Accuracy	0.41	72
Williams	2014	Tower of Hanoi	Total moves	0.24	130
Williams	2012	Tower of London	Total moves	0.26	34
Williams & Jarrold	2013	Tower of London	Total moves	0.59	43
Zinke	2010	Tower of London	Total perfect solutions	0.98	32

© 2019 Rødgaard E-M et al. *JAMA Psychiatry.*

eTable 5. Data for Inhibition (Autism)

Title	Year	Type	Outcome	Effect	Participants
Adamo	2013	Go-No-Go	Commission errors	0.33	82
Adams & Jarrold	2009	Stroop	RT interference	-0.39	24
Adams & Jarrold	2012	Stop	comission errors	0.3	48
Adams and Jarrold	2012	Flanker	RT incongruent	-0.28	30
Ambrosino	2014	Go-No-Go	% correct trials	0.43	38
Andersen	2015	Stroop	RT condition 4	0.94	79
Barron-Linnankoski	2015	Stroop	Switching score	-0.14	90
Bishop	2005	WDW	Number of correct responses	1.15	32
Bishop	2005	Opposite World	Time difference	1.35	29
Brandimonte	2011	Go-No-Go	% correct trials	0.34	20
Chan (a)	2011	Stroop	Number of errors	0.35	40
Chan (b)	2014	Continuous Performance	Commission errors	0.005	38
Chan (b)	2011	Go-No-Go	Commission errors	0.59	40
Chien	2014	Continuous Performance	Commission errors	0.43	496
Christ	2007	Go-No-Go	Commission errors	0.93	43
Christ	2007	Flanker	RT incongruent	1.93	43
Christ	2011	Flanker	% incongruent	0.6	77
Corbett	2009	Stroop	RT condition 4	1.46	36
Czemainski	2014	Stroop	SCW score	0.69	30
Dichter and Belger	2008	Flanker	RT incongruent	-0.76	34
Geurts	2008	Flanker	RT incongruent	0.06	44
Geurts	2004	Opposite World	Time difference	0.37	82
Geurts	2004	Stop	SSRT	0.73	82
Geurts	2009	Go-No-Go	Commission errors	0.42	40
Goddard	2014	Junior Hayling Test	Score	-0.03	102
Goddard	2014	Stroop	Total score of section B	0.41	126
Goldberg	2005	Stroop	SCW score	0.11	49
Happé	2006	Go-No-Go	Commission errors	-0.19	64
Henry	2014	Stroop	RT condition 4	0.1	60
Jahromi	2013	Stroop	Nr correct trials	0.34	40
Johnston	2011	Stroop	Number of errors	0.11	38
Kilincaslan	2010	Continuous Performance	Commission errors	0.61	43
Kilincaslan	2010	Stroop	Correct responses	0.11	39

© 2019 Rødgaard E-M et al. *JAMA Psychiatry.*

Kretschmer	2014	Go-No-Go	% correct trials	0.48	59
Langen	2011	Go-No-Go	Correct No-Go	1.17	43
Larson	2012	Flanker	RT incongruent	0.07	64
Lee	2009	Go-No-Go	Commission errors	0.26	24
Lemon	2011	Stop	SSRT	0.52	45
Mahone	2006	Luria Hand Game	Number of correct	-0.051	57
Maister (a)	2013	Stroop	Correct responses	-0.18	28
Narzisi	2013	Stroop	Switching score	1.23	66
Ozonoff	1994	Go-No-Go	Commission errors	0.05	28
Ozonoff & Strayer	1997	Stop	SSRT	0.51	26
Ozonoff and Jensen	1999	Stroop	NR	0.41	69
Pankert	2014	Go-No-Go	Commission errors	0.72	34
Pellicano	2006	Luria Hand Game	Number of correct	0.67	66
Perez	2009	Stroop	Number of errors	0.94	31
Robinson	2009	Stroop	Correct responses	0.3	108
Robinson	2009	Junior Hayling Test	Score	0.43	84
Russell	1999	Stroop	RT incongruent	0.36	38
Samyn	2015	Go-No-Go	Commission errors	0.46	179
Samyn	2015	Stroop	Correct responses	0.073	179
Sanderson and Allen	2012	Go-No-Go	Commission errors	-0.68	59
Schmitz	2006	Go-No-Go	Number of incorrect trials	0.25	22
Semrud-Clikeman	2010	Stroop	RT condition 4	0.47	47
Sinzig	2014	Go-No-Go	Commission errors	0.78	56
Sinzig	2008	Go-No-Go	Commission errors	0.17	40
South	2010	Flanker	RT incongruent	-0.5	45
Terrett	2013	Stroop	Switching score	-0.12	60
Tye	2014	Continuous Performance	Commission errors	0.32	74
Vara	2014	Go-No-Go	Commission errors	0.62	30
Verte	2006	Opposite World	Time difference	0.35	139
Voelbel	2006	Stroop	SCW score	0.85	51
Weissman	2010	Stroop	SCW score	0.41	82
Xiao	2012	Go-No-Go	Commission errors	0.87	35
Xiao	2012	Stroop	RT incongruent	0.2	35
Yasumura	2014	Stroop	Correct responses	-0.09	26
Yerys (a)	2009	WDW	Score	0.45	70
Yoran-Hegesh	2009	Stroop	Correct responses	0.22	66
Zandt	2009	WDW	Score	0.4	37
van Eylen	2015	Go-No-Go	Commission errors	0.5	100

© 2019 Rødgaard E-M et al. *JAMA Psychiatry.*

eTable 6. Data for P3b Amplitude (Autism)

Title	Year	Type	Effect	Participants
Andersson et al. (2013)	2013	Auditory	0.28	23
Ciesielski et al.	1990	Both	0.59	23
Clery et al. (2)	2013	Visual	-0.54	24
Courchesne et al. (1984)	1984	Auditory	2.66	14
Courchesne et al. (1989)	1989	Both	0.93	27
Erwin et al.	1991	Auditory	0.11	25
Kohls et al.	2011	Visual	0.44	36
Lincoln et al.	1993	Auditory	1.33	18
Novick et al.	1980	Auditory	1.82	10
Oades et al.	1988	Auditory	1.84	16
Salmond et al.	2007	Auditory	0.45	45
Senju et al.	2005	Visual	0.01	28
Tye et al.	2014	Visual	0.28	45
Verbaten et al.	1991	Visual	0.72	40

© 2019 Rødgaard E-M et al. *JAMA Psychiatry.*

Table 7. Data for Brain Size (Autism)

Title	Year	Type	Effect	Participants
Akshoomoff (a)	2004	Brain volume (MRI)	5.5	45
Akshoomoff (b)	2004	Brain volume (MRI)	4.92	27
Akshoomoff (c)	2004	Brain volume (MRI)	5.66	25
Aylward	1999	Brain volume (MRI)	1.58	28
Aylward (a)	2002	Brain volume (MRI)	1.959	51
Aylward (b)	2002	Brain volume (MRI)	1.22	47
Aylward (c)	2002	Brain volume (MRI)	1.05	52
Bailey	1995	Head circumference	23.41	21
Bigler	2010	Brain volume (MRI)	1.63	101
Bloss (a)	2007	Brain volume (MRI)	7.94	23
Bloss (b)	2007	Brain volume (MRI)	5.25	40
Bolton	1994	Head circumference	18.99	27
Calderoni	2012	Brain volume (MRI)	2.56	76
Carper (a)	2002	Brain volume (MRI)	38.259	20
Carper (b)	2002	Brain volume (MRI)	1.56	36
Carper (c)	2002	Brain volume (MRI)	2.22	21
Cederlund	2014	Head circumference	1	33
Chaste	2013	Head circumference	5.57	1889
Chawarska	2011	Head circumference	8.59	98
Cheung	2011	Brain volume (MRI)	1.19	91
Cleavinger	2008	Brain volume (MRI)	1.56	44
Courchesne (a)	2001	Brain volume (MRI)	11.986	42
Courchesne (b)	2001	Brain volume (MRI)	1.259	29
Courchesne (c)	2001	Brain volume	1.35	24

© 2019 Rødgaard E-M et al. *JAMA Psychiatry.*

		(MRI)		
Davidovitch	2011	Head circumference	1.49	317
Davidovitch	1996	Head circumference	7.19	148
Dementieva	2005	Head circumference	7.58	251
Deutsch & Joseph	2003	Head circumference	5.26	63
Fidler	2000	Head circumference	4.49	41
Fombonne	1999	Head circumference	6.48	126
Freitag	2009	Brain volume (MRI)	1.47	30
Froehlich	2013	Head circumference	8.59	255
Fuller Torrey	2004	Head circumference	4.96	15
Ghaziuddin	1999	Head circumference	8.08	20
Gillberg (a)	2002	Head circumference	3.2	50
Gillberg (b)	2002	Head circumference	8.54	50
Girgis	2007	Brain volume (MRI)	1.42	29
Grandgeorge	2013	Head circumference	1.95	422
Greimel	2013	Brain volume (MRI)	2.32	98
Griebling	2010	Brain volume (MRI)	1.62	70
Hallahan (a)	2009	Brain volume (MRI)	1.097	174
Hallahan (b)	2009	Brain volume (MRI)	1.094	140
Hallahan (c)	2009	Brain volume (MRI)	1.51	88
Hallahan (d)	2009	Brain volume (MRI)	2.08	66
Hardan	2009	Brain volume (MRI)	1.42	45
Hardan	2003	Brain volume (MRI)	1.64	81
Hardan	2008	Brain volume (MRI)	1.22	24
Hardan	2000	Brain volume (MRI)	2.05	35
Haznedar	2000	Brain volume	1.13	34

© 2019 Rødgaard E-M et al. *JAMA Psychiatry.*

		(MRI)		
Herbert	2003	Brain volume (MRI)	3.56	32
Hong	2011	Brain volume (MRI)	1.52	34
Jou	2010	Brain volume (MRI)	1.45	37
Jou (a)	2010	Brain volume (MRI)	2.33	14
Jou (b)	2010	Brain volume (MRI)	3.54	17
Kates	2004	Brain volume (MRI)	2.15	25
Lainhart	1997	Head circumference	5.4	91
Lainhart	2006	Head circumference	6.76	338
McAlonan	2002	Brain volume (MRI)	1.37	45
Miles	2000	Head circumference	9.88	137
Miles	2008	Head circumference	6.62	172
Mostofsky	2007	Brain volume (MRI)	1.21	56
Nordahl	2013	Brain volume (MRI)	2.72	171
Nur Say	2014	Brain volume (MRI)	1.32	30
Palmen	2004	Brain volume (MRI)	3.12	42
Palmen	2005	Brain volume (MRI)	4.196	42
Pierce & Courchesne	2001	Brain volume (MRI)	1.44	28
Piven	1995	Brain volume (MRI)	4.17	42
Rojas	2002	Brain volume (MRI)	6.81	30
Sacco	2006	Head circumference	14.59	241
Schumann	2010	Brain volume (MRI)	4.21	85
Schumann (a)	2004	Brain volume (MRI)	1.65	40
Schumann (b)	2004	Brain volume (MRI)	1.62	43
Schumann (c)	2004	Brain volume (MRI)	1.299	46
Scott	2009	Brain volume	1.05	62

		(MRI)		
Skjeldal	1998	Head circumference	4.41	25
Sparks	2002	Brain volume (MRI)	6.81	71
Stamova	2013	Brain volume (MRI)	3.51	50
Stevenson	1997	Head circumference	10.21	100
Tamura (a)	2010	Brain volume (MRI)	4.04	28
Tamura (b)	2010	Brain volume (MRI)	1.24	31
Tamura (c)	2010	Brain volume (MRI)	1.189	27
Tate	2007	Brain volume (MRI)	1.54	60
Tepest	2010	Brain volume (MRI)	1.11	58
Tsatsanis	2003	Brain volume (MRI)	1.57	24
Van Daalen	2007	Head circumference	4.12	53
Ververi	2012	Head circumference	8.7	222
Vidal	2006	Brain volume (MRI)	1.23	50
Webb	2007	Head circumference	8.8	28
Woodhouse	1996	Head circumference	13.66	37

© 2019 Rødgaard E-M et al. *JAMA Psychiatry.*

eTable 8. Data for Theory of Mind (Schizophrenia)

Title	Year	Type	Effect	Participants
Ba et al.	2008	False belief 2	1.92	32
Ba et al.	2008	False belief 1	0.73	32
Bertrand et al.	2007	Hints	1.09	63
Bonshtein et al.	2006	False belief 1	1.25	63
Bonshtein et al.	2006	False belief 2	1.7	63
Brune	2003	False belief	0.86	35
Brune and Bodenstein	2005	FB-seq	1.52	52
Brune and Bodenstein	2005	False belief	1.64	52
Brune et al.	2007	FB-seq	1	67
Brune et al.	2007	False belief	0.97	67
Brunet et al.	2003	Character Intent Interference	0.88	50
Corcoran et al.	2008	FB-seq	0.69	92
Corcoran et al.	2008	False belief 2	0.62	85
Corcoran et al.	1995	Hints	0.82	85
Corcoran et al.	2003	Hints	0.99	103
Craig	2004	Hints	1.58	32
Gavilan et al.	2011	Strange Stories	1.8	44
Harrington et al.	2005	False belief 1	0.55	63
Harrington et al.	2005	FB-seq	0.65	63
Harrington et al.	2005	False belief 2	0.73	63
Herold et al.	2009	Faux Pas	0.82	39
Hooker et al.	2011	Faux Pas	1.46	38
Langdon et al.	2001	FB-seq	1.23	56
Langdon et al.	2002	FB-seq	1.54	45
Langdon et al.	2002	Irony	1.28	45
Langdon et al.	1997	FB-seq	0.75	40
Langdon et al.	2006	FB-seq	1.41	55
Marjoram	2005	Hints	2.13	30
Martino et al.	2007	Faux Pas	1.3	36
Mo et al.	2008	Irony	1.41	51
Pickup & Frith	2001	False belief	1.61	76
Pijnenborg et al.	2009	Faux Pas	0.68	99
Pinkhan & Penn	2006	Hints	0.62	93
Pinkhan and Penn	2006	False belief	0.62	93
Pousa et al.	2008	FB-seq	0.1	112
Pousa et al.	2008	False belief 2	0.25	112
Randall et al.	2003	False belief 2	1.58	50
Randall et al.	2003	False belief 1	0.93	50
Riveros et al.	2010	Faux Pas	0.92	33
Sarfati et al.	1997	Character Intent Interference	1.31	48

© 2019 Rødgaard E-M et al. *JAMA Psychiatry.*

Sarfati et al.	1999	Character Intent Interference	1.11	40
Shur et al.	2008	Faux Pas	0.9	61
Stanford et al.	2011	Strange Stories	1.03	27
Tsoi et al.	2008	FB-seq	1.11	60
Zalla et al.	2004	FB-seq	2	80
Zhu et al.	2007	Faux Pas	1.27	71
de Achával et al.	2010	Faux Pas	0.84	40

© 2019 Rødgaard E-M et al. *JAMA Psychiatry.*

eTable 9. Data for Stroop Task (Schizophrenia)

Title	Year	Type	Effect	Participants
Barch	2005	Mix	1.1	32
Barch	2005	Computer	0.41	32
Barch	2004	Computer	0.35	58
Barch	2004	Mix	0.26	58
Barch (a)	1999	Mix	0.8	60
Barch (a)	1999	Computer	0.22	60
Barch (b)	1999	Mix	0.97	81
Barch (b)	1999	Computer	-0.17	81
Barr	2008	Card	0.95	60
Boucart	1999	Computer	-0.32	24
Brebion	1996	Card	0.95	64
Breton	2011	Card	0.85	105
Buchanan	1994	Card	0.73	69
Carter	1997	Mix	0.97	29
Carter	1993	Computer	0.23	37
Carter	1997	Computer	0.04	29
Chen	2001	Computer	0.07	120
Chen	2001	Mix	0.17	120
Dollfus	2002	Card	-0.46	34
George	2002	Computer	0.97	60
Golden	1976	Card	-0.32	72
Haker	2009	Card	0.47	88
Henik	2002	Mix	0.65	27
Henik	2002	Computer	-0.11	27
Hepp	1996	Computer	0.77	94
Hepp	1996	Card	0.32	94
Hepp	1996	Mix	0.57	94
Jaquet	1997	Mix	0.57	61
Jaquet	1997	Card	0.71	61
Killian	1984	Card	1.07	60
Killian	1984	Mix	0.98	60
Markela_Lerenc	2009	Computer	0.44	30
Matsuzawa	2008	Card	0.97	36
McGowan	2004	Card	-0.07	28
McNeely	2003	Computer	0.58	26
Moritz	2002	Card	1.41	95
Mulet	2007	Card	1.37	85
Nordahl	2001	Computer	0.42	19
Perlstein	1998	Mix	0.9	79
Perlstein	1998	Computer	-0.16	79
Perlstein	1998	Card	-0.48	79
Rizzo	1996	Card	0.57	66
Rizzo	1996	Mix	0.5	66

© 2019 Rødgaard E-M et al. *JAMA Psychiatry.*

Sacco	2006	Computer	0.22	29
Salo	1997	Computer	-0.23	40
Salo	2002	Computer	0.05	39
Scholes	2010	Card	0.51	84
Szoke	2009	Card	0.6	96
Takei	2009	Computer	-0.05	96
Taylor	1996	Computer	0.13	24

© 2019 Rødgaard E-M et al. *JAMA Psychiatry.*

eTable 10. Data for Grey Matter Volume (Schizophrenia)

”Grey matter volume” was chosen for analysis over the more closely matching ”total brain volume”, as data for the latter was not provided in a readable format.

Title	Year	Type	Effect	Participants
Anath	2002	GM	1.228	40
Andreone	2007	GM	0.666	50
Baare	1999	GM	0.361	27
Bodnar	2010	GM	0.611	74
Bodnar	2010	GM	0.082	97
Boonstra	2011	GM	0.346	36
Bose	2009	GM	0.921	67
Brown	2011	GM	0.742	38
Crespo-Facoro	2009	GM	0.265	165
Delcken	2002	GM	0.781	80
Ganeshan	2010	GM	1.053	32
Gur	1999	GM	0.284	110
Gur	1999	GM	0.649	150
Ha	2005	GM	0.94	71
Hasan	2011	GM	-0.138	46
Ho	2007	GM	1.148	92
Horn	2010	GM	0.852	40
Hubl	2010	GM	-0.164	26
Hubl	2010	GM	0.392	24
Hulshoff	2002	GM	0.189	317
Hulshoff	2004	GM	0.444	22
Hulshoff	2004	GM	0.675	22
James	2011	GM	0.651	44
James	2011	GM	0.828	44
Jang	2006	GM	0.15	46
Kumra	2011	GM	0.618	100
Meisenzah	1999	GM	0.188	46
Molina	2005	GM	0.991	23
Morgan	2007	GM	0.24	88
Moriya	2010	GM	0.428	38
Naravan	2007	GM	0.816	42
Narr	2005	GM	0.068	88
Narr	2005	GM	0.588	62
Narr	2003	GM	0.389	23
Narr	2003	GM	0.18	30
O'Daly	2007	GM	0.336	60
Okugawa	2007	GM	0.335	57
Okugawa	2007	GM	0.616	138
Ortiz	2011	GM	1.107	65
Ortiz	2011	GM	0.743	62
Pagsberg	2007	GM	0.393	44

© 2019 Rødgaard E-M et al. *JAMA Psychiatry.*

Premkumar	2009	GM	0.471	50
Premkumar	2009	GM	0.55	44
Prestia	2011	GM	1.577	39
Rameti	2010	GM	0.158	46
Reig	2011	GM	0.143	85
Rossell	2001	GM	0.125	60
Rossell	2001	GM	0.252	73
Rusch	2007	GM	0.761	92
Rusch	2007	GM	1.138	83
Schiffer	2010	GM	-0.069	25
Schiffer	2010	GM	0.282	26
Sigmundson	2001	GM	0.469	54
Sporn	2003	GM	0.347	82
Suzuki	2005	GM	0.462	112
Takahashi	2009	GM	0.528	108
Takao	2010	GM	0.411	96
Tanskanen	2009	GM	0.261	154
Thoma	2008	GM	0.383	44
Ueda	2010	GM	0.392	123
Voeds	2008	GM	0.712	50
Yoon	2005	GM	0.408	118
Yoshihara	2008	GM	0.755	36

© 2019 Rødgaard E-M et al. *JAMA Psychiatry.*

eTable 11. List of Excluded Primary Studies

For some studies, several effect sizes were listed in meta-analyses. Some exclusions only regard one of the listed effect sizes. Individual studies can thus be present in the table below as well as one of Tables 1-10.

**Study**	**Construct**	**Meta-analysis**	**Change**	**Reason**
Kleinman et al (2001)	Emotion recognition	Leppanen et al	Publication year corrected	Incorrectly reported as 2000
David et al (2008)	Theory of mind	Chung et al	Effect size	Incorrectly calculated
Oakley et al. (2016)	Emotion recognition	Leppanen et al. (2018)	Excluded	Control group is matched on alexithymia traits, which affects outcome
Eack et al. (2013)	Cognitive flexibility	Westwood et al. (2016)	Excluded	The effect size pertaining to persevative errors in Eack et al. (2013) table 2 is not consistent with the value calculated with their mean values.
Pooragha et al. (2013)	Cognitive flexibility	Westwood et al. (2016)	Excluded	Different criteria for inclusion for autism at least (80) full scale IQ and control group at least (90) full scale IQ
Golan et al. 2006	Emotion recognition	Leppanen et al. (2018)	Excluded	Only study using RMFT
Rosenblau et al. (2015)	Emotion recognition	Leppanen et al. (2018)	Excluded	Only study using the AoE test
Schaller & Rauh (2017)	Emotion recognition	Leppanen et al. (2018)	Excluded	Only study using the A-ToM test
Shamay-Tsoory (2008)	Emotion recognition	Leppanen et al. (2018)	Excluded	Only study using the C-ToM test
Dziobek et al. (2006b)	Emotion recognition	Leppanen et al. (2018)	Excluded	Only study using the MASC - Emotions test
Heavey et al. (2000)	Theory of mind	Leppanen et al. (2018)	Excluded	Only study using the AMT test
Schaller & Rauh (2017)	Theory of mind	Leppanen et al. (2018)	Excluded	Only study using the A-ToM test
Callenmark et al. (2014)	Theory of mind	Leppanen et al. (2018)	Excluded	Only study using the DST test
Craig et al. (2004)	Theory of mind	Leppanen et al. (2018)	Excluded	Only study using the Hint task
Schneider et al. (2013)	Theory of mind	Leppanen et al. (2018)	Excluded	Only study using the ToM scale test

© 2019 Rødgaard E-M et al. *JAMA Psychiatry.*

Samson & Hegenloh (2010)	Theory of mind	Leppanen et al. (2018)	Excluded	Only study using the Cartoons test
Beaumont & Newcombe (2006)	Theory of mind	Leppanen et al. (2018)	Excluded	Only study using the Commercials test
Shamay-Tsoory (2008)	Theory of mind	Leppanen et al. (2018)	Excluded	Only study using the C-ToM test
Martin & McDonald	Theory of mind	Leppanen et al. (2018)	Excluded	Only study using the PIS test
Blackshaw et al. (2001)	Theory of mind	Leppanen et al. (2018)	Excluded	Only study using the PIT test
Begeer et al. (2010)	Theory of mind	Leppanen et al. (2018)	Excluded	Only study using the Reading task
Beaumont & Newcombe (2006)	Theory of mind	Leppanen et al. (2018)	Excluded	Only study using the TAT test
Bennetto et al. (1996)	Cognitive Flexibility	Landry & Al-Taie (2016)	Excluded	Invalid control group
Bramham et al. (2009)	Planning	Olde Dobbelink & Geurts (2017)	Excluded	No reported effect size (key Search test)
Brunsdon et al. (2015)	Planning	Olde Dobbelink & Geurts (2017)	Excluded	Only study using Planning drawing task, Part B (planning)
Hanson & Atance	Planning	Olde Dobbelink & Geurts (2017)	Excluded	Only study using the Truck loading test
Hill & Bird (2006)	Planning	Olde Dobbelink & Geurts (2017)	Excluded	No reported effect size (key Search test)
Hughes et al. (1994)	Planning	Olde Dobbelink & Geurts (2017)	Excluded	Only study in Stockings of Cambridge that use Decision time as effect meassure
Lopez et al. (2005)	Planning	Olde Dobbelink & Geurts (2017)	Excluded	Only study using Tower of California
Pellicano et al. (2006)	Planning	Olde Dobbelink & Geurts (2017)	Excluded	No reported effect size (Tower of London)
Pellicano et al. (2007)	Planning	Olde Dobbelink & Geurts (2017)	Excluded	No reported effect size (Tower of London)
Rajendran et al. (2011)	Planning	Olde Dobbelink & Geurts (2017)	Excluded	No reported effect size (key Search test)
Rajendran et al. (2005)	Planning	Olde Dobbelink & Geurts (2017)	Excluded	Only study using the Six Elements test

© 2019 Rødgaard E-M et al. *JAMA Psychiatry.*

Taddei & Contena (2013)	Planning	Olde Dobbelink & Geurts (2017)	Excluded	Only study using the Cognitive Assessment System (CAS) - Planning
White et al (2009)	Planning	Olde Dobbelink & Geurts (2017)	Excluded	No reported effect size (key Search test)
Williams et al. (2014)	Planning	Olde Dobbelink & Geurts (2017)	Excluded	No reported effect size (key Search test)
Prior & Hoffmann (1990)	Planning	Olde Dobbelink & Geurts (2017)	Excluded	Only study using the Milner Mazes task
Davies et al 1994, effect size 1	Emotion recognition (Ekman)	Uljarevic & Hamilton (2013)	Excluded	Uses their own test, not an Ekman test.
Courchesne et al. (1985)	P3b amplitude	Cui et al. (2017)	Excluded	Effect size listed in Cui et al. is opposite what is described by the study
Spezio et al. (2007)	Emotion recognition (Ekman)	Uljarevic & Hamilton (2013)	Excluded	Variation of Ekman test not used by other studies represented in meta-study
Tantam et al. (1989)	Emotion recognition (Ekman)	Uljarevic & Hamilton (2013)	Excluded	Variation of Ekman test not used by other studies represented in meta-study
Buitelaar et al. (1999)	Emotion recognition (Ekman)	Uljarevic & Hamilton (2013)	Partially excluded	Reports effect sizes for two variants of Ekman test on the same sample. ES for non-standard variant is excluded
Baron-Cohen et al. (1997)	Emotion recognition	Leppanen et al. (2018)	Effect size	Two effect sizes were reported. IQ data age and effect sizes suggests that both use the same sample. The mean of the two effect sizes was used
Williams et al. (2013)	Cognitive flexibility	Lai et al. (2017)	Effect size	IQ data age and effect sizes suggests that the used sample is a duplicate of Williams & Jarrold (2013). The mean of the two effect sizes was used

© 2019 Rødgaard E-M et al. *JAMA Psychiatry.*

eTable 12. NOS Ratings for Emotion Recognition

The Newcastle-Ottawa Scale (NOS) for Assessing the Quality of Studies Included - AUTISM adaptation																	
		Selection (Tot = 4)				Comparability (Tot=2)	Outcome (Tot=3)			NOS Total	n autistic group	n control group	Autism group composition	Syn-dromic autism	IQ autistic group	IQ control group	IQ TEST
	item #	1	2	3	4	5	6	7	8								
Emotion recognition																	
Adler et al.	2010	0	0	0	0.0	1.0	1.0	1.0	1.0	4.0	16	21	0	0	NA	NA	WAIS
David et al.	2008	0.0	1.0	0.0	1.0	2.0	1.0	1.0	1.0	7.0	24	24	0	0	130.1	135.7	WAIS
Dziobek et al	2006	1.0	0.0	1.0	1.0	2.0	1.0	1.0	1.0	8.0	19	20	0	0	122.0	124.0	WAIS
Spek et al.	2010	1.0	1.0	1.0	1.0	2.0	1.0	1.0	1.0	9.0	61	32	0	1	112.4	115.9	WAIS
Baron-Cohen et al.	2001	0.0	1.0	1.0	0.0	2.0	1.0	1.0	1.0	7.0	15	14	0	0	115.0	116.0	WAIS
Craig et al.	2004	0.0	0.0	1.0	0.0	2.0	1.0	1.0	1.0	6.0	17	16	0	0	104.8	110.3	NART
Couture et al.	2010	0.5	1.0	0.0	1.0	1.0	1.0	1.0	1.0	6.5	36	41	1	1	101.3	109.4	WASI
Gonzalez-Gadea et al	2013	0.0	NA	1.0	0.0	2.0	1.0	1.0	1.0	6.0	23	21	0	0	NA	NA	NA
Lahera et al	2014	1.0	NA	0.0	1.0	1.0	1.0	1.0	1.0	6.0	22	26	0	0	NA	NA	NA
Lugnegard et al	2013	0.0	0.0	0.0	1.0	2.0	1.0	1.0	1.0	6.0	53	50	0	0	10.4	9.9	WAIS Vocabulary
Demurie et al	2011	1.0	1.0	0.0	0.0	1.0	1.0	1.0	1.0	6.0	13	18	0	0	105.2	NA	NA
Kritsten et al	2014	1.0	1.0	1.0	1.0	2.0	1.0	1.0	1.0	9.0	20	20	0	0	100.8	103.9	Nonverbal IQ
Murray et al	2017	1.0	1.0	1.0	1.0	2.0	1.0	1.0	1.0	9.0	20	20	0	1	105.1	111.3	Verbal IQ
Robinson et al	2017	0.0	0.0	1.0	1.0	2.0	1.0	1.0	1.0	7.0	24	24	0	0	104.3	103.6	WASI
Rosenblau et al	2015	0.5	1.0	1.0	1.0	2.0	1.0	1.0	1.0	8.5	28	23	0	0	113.0	108.0	Verbal IQ (MWT)
Schaller & Rauh	2017	0.5	0.0	1.0	1.0	2.0	1.0	1.0	1.0	7.5	23	22	0	0	105.7	103.8	Nonverbal IQ (CFT 20-R)
Schuwerk et al	2015	0.0	1.0	1.0	1.0	2.0	1.0	1.0	1.0	8.0	18	19	0	0	91.4	98.3	Nonverbal IQ (CFT 20-R)
Lombardo	2007	0.0	1.0	0.0	1.0	2.0	1.0	1.0	1.0	7.0	30	30	0	0	117.2	117.1	WASI
Muller	2016	1.0	1.0	0.0	0.0	1.0	1.0	1.0	1.0	6.0	33	23	0	0	101.1	109.8	WISC
Sato	2017	1.0	1.0	1.0	1.0	2.0	1.0	1.0	1.0	9.0	19	19	0	0	112.3	114.8	WAIS

© 2019 Rødgaard E-M et al. *JAMA Psychiatry.*

Segura	2015	1.0	1.0	0.0	1.0	1.0	1.0	1.0	1.0	7.0	21	10	0	0	102.0	110.0	WAIS/WISC
Wilson	2014	0.5	1.0	1.0	1.0	2.0	1.0	1.0	1.0	8.5	89	89	0	1	110.0	114.0	WASI
Baron-Cohen	2015	0.0	NA	0.0	1.0	1.0	1.0	1.0	1.0	5.0	395	320	0	0	NA	NA	NA
Lai	2012	1.0	1.0	1.0	1.0	2.0	1.0	1.0	1.0	9.0	64	64	0	1	113.9	118.0	NA
Pedreno	2017	1.0	1.0	0.0	1.0	1.0	1.0	1.0	1.0	7.0	35.0	35.0	0	0	100.0	115.2	WISC/WAIS
Golan et al.	2007	0.0	1.0	1.0	1.0	2.0	1.0	1.0	1.0	8.0	50.0	22.0	0	0	113.8	114.5	WASI
Kleinman et al.	2001	0.5	NA	0.0	0.0	0.0	1.0	1.0	0.0	2.5	24.0	24.0	1	0	NA	NA	NA
Torralva et al	2013	0.0	NA	0.0	0.0	1.0	1.0	1.0	1.0	4.0	25.0	25.0	0	0	NA	NA	NA
Baron-Cohen et al.	1997	0.0	1.0	1.0	1.0	2.0	1.0	1.0	1.0	8.0	16.0	16.0	0		105.3	100.0	WAIS and NART Two different IQ tests?
Golan et al.	2007	0.0	1.0	1.0	1.0	2.0	1.0	1.0	1.0	8.0	50.0	22.0	0	0	113.8	114.5	WASI
Rutherford	2002	1.0	1.0	1.0	1.0	1.0	1.0	1.0	1.0	8.0	19.0	20.0	0	0.0	107.9	101.0	WAIS
Kleinman et al.	2001	0.5	NA	0.0	0.0	0.0	1.0	1.0	0.0	2.5	24.0	24.0	1	0	NA	NA	NA
Roeyers et al. Rewritten SS	2001	0.0	1.0	1.0	1.0	1.0	1.0	1.0	1.0	7.0	24.0	24.0	0	0.0	113.0	NA	WAIS
Ponnet et al. rewritten SS	2004	0.0	1.0	0.0	0.0	2.0	1.0	1.0	1.0	6.0	19.0	19.0	0	0.0	106.6	114.1	WAIS-R
Lehnhardt et al.	2011	1.0	1.0	1.0	1.0	2.0	1.0	1.0	1.0	9.0	39.0	39.0	0	0.0	127.9	133.3	WAIS-German
Brent et al.	2004	0.0	1.0	0.0	1.0	1.5	1.0	1.0	1.0	6.5	20.0	20.0	0	0.0	99.8	105.9	WISC
Holt et al.	2014	0.5	1.0	0.0	1.0	1.0	1.0	1.0	1.0	6.5	49.0	40.0	0	0.0	103.4	112.4	WASI
Kaland et al.	2008	1.0	1.0	0.0	1.0	1.0	1.0	1.0	1.0	7.0	21.0	20.0	0	0.0	106.4	122.7	WISC
Kirchner et al.	2011	1.0	0.0	0.0	0.0	2.0	1.0	1.0	1.0	6.0	20.0	21.0	1	0.0	112.6	110.1	Vocabulary test
Otsuka et al.	2017	1.0	0.0	1.0	0.0	2.0	1.0	1.0	1.0	7.0	21.0	21.0	0	1.0	112.0	113.6	WAIS
Peterson et al.	2015	0.0	1.0	1.0	1.0	1.5	1.0	1.0	1.0	7.5	34.0	41.0	0	0.0	93.2	103.6	PPVT-R
Rueda et al.	2015	0.0	0.0	1.0	0.0	2.0	1.0	1.0	1.0	6.0	38.0	38.0	0	0.0	102.0	106.7	WISC
Vogindroukas	2014	0.0	0.0	0.0	1.0	0.5	1.0	1.0	1.0	4.5	27.0	53.0	1	0.0	93.6	NA	WISC
Baron-Cohen et al.	1999	0.0	0.0	1.0	0.0	2.0	1.0	1.0	1.0	6.0	6.0	12.0	0	0.0	108.5	110.0	WAIS
Braverman	1989	0.0	NA	1.0	0.0	2.0	1.0	0.0	1.0	5.0	15.0	30.0	0	0.0	NA	NA	NA
Braverman	1989	0.0	NA	1.0	0.0	2.0	1.0	1.0	1.0	6.0	15.0	30.0	0	0.0	NA	NA	NA

© 2019 Rødgaard E-M et al. *JAMA Psychiatry.*

Macdonald	1989	0.0	1.0	1.0	0.0	2.0	1.0	1.0	1.0	7.0	10.0	10.0	1	1.0	118.4	120.1	Raven
Tantam	1989	0.0	1.0	1.0	1.0	2.0	1.0	1.0	1.0	8.0	10.0	10.0	1	0.0	NA	NA	NA
Davies	1994	0.0	1.0	1.0	1.0	2.0	1.0	1.0	1.0	8.0	10	10.0	0	0.0	84.9	88.1	Raven
Buitelaar	1999	0.5	1	0	1.0	1	1	1	1	6.5	20	20	1	0	102.1	NA	WISC-R
Celani	1999	0.0	1.0	1.0	0.0	1.0	1.0	1.0	1.0	6.0	10.0	10.0	1	0.0	63.2	101.6	NA
Grossman	2000	0	1	1	1	2	1	1	1	8.0	13	13	0	0	106.4	116.2	WISC
Pelphrey	2002	0.5	0	0	1	1	1	1	1	5.5	5	5	1	0	100.75	NA	WAIS-R
Bolte & Poustka	2003	1	1	0	1	2	1	1	1	8.0	15	22	1		103.7	112.9	Raven
Piggot	2004	1	1	1	1	2	1	1	1	9.0	14	10	0	1	112	116	WASI
Piggot	2004	1	1	1	1	2	1	1	1	9.0	14	10	0	1	112	116	WASI
Castelli	2005	0	1	0	0	1.5	1	1	1	5.5	20	20	0	0	NA	NA	NA
Dziobek	2006	1	0	1	1	2	1	1	1	8.0	17	17	0	0	113	115	Shipley IQ/WAIS
Boraston	2007	0.5	1	1	1	2	1	1	1	8.5	11	11	0	0	117	114	WASI
Clark	2008	1	1	0	1	1.5	1	1	1	7.5	15	21	0	1	99.5	109.4	PPVT
Corden	2008	1	0	1	0	2	1	1	1	7.0	21	21	0	0	117.9	117.2	WASI
Wright	2008	1	1	1	1	2	1	1	1	9.0	35	35	0	0	104.63	103.86	WASI
Phillip	2010	1	1	0	1	1	1	1	1	7.0	23	23	0	0	101.5	111.2	WASI
Jones	2011	0.5	1	0	0	2	1	1	1	6.5	97	55	0	0	90.6	91.5	WASI
Wallace, Coleman & Bailey expt 1	2008	1	0	1	1	2	1	1	1	8.0	26	26	0	1	122	117	Wechsler

© 2019 Rødgaard E-M et al. *JAMA Psychiatry.*

eTable 13. NOS Ratings Theory of Mind

The Newcastle-Ottawa Scale (NOS) for Assessing the Quality of Studies Included - AUTISM adaptation																		
		Selection (Tot = 4)				Comparability (Tot=2)	Outcome (Tot=3)			NOS Total	n autistic group	n control group	Autism group composition	Syndromic autism	IQ autistic group	IQ control group	IQ TEST
	item #	1	2	3	4	5	6	7	8								
Theory of mind (meta-analysis from Leppanen et al., 2018)																	
Beversdorf	1998	0.5	1.0	1.0	0.0	2.0	1.0	1.0	1.0	7.5	10	13	0	0	109.7	117.3	FSIQ WAIS-R
Bowler	1992	0.0	0.0	0.0	1.0	0.0	1.0	1.0	1.0	4.0	15	15	0	0	86.8	NA	FSIQ WAIS
Brewer	2017	0.0	1.0	1.0	1.0	1.5	1.0	1.0	1.0	7.5	163	80	0	0	108.7	106.4	PRI WAIS
Brown & Klein	2011	0.0	0.0	1.0	0.0	2.0	1.0	1.0	1.0	6.0	16	16	0	0	12.6	14.2	
Brunsdon	2015	1.0	1.0	1.0	1.0	2.0	1.0	1.0	0.0	8.0	181	160	0	0	90.0	101.9	mix WASI
Craig et al.	2004	0.0	0.0	1.0	0.0	1.5	1.0	1.0	1.0	5.5	17	16	0	0	104.76	110.25	NART
Crane	2011	0.0	1.0	1.0	0.0	2.0	1.0	1.0	1.0	7.0	28	28	0	0	117.2	115.1	FSIQ WASI
Dziobek (a)	2006	1.0	0.0	1.0	1.0	2.0	0.0	1.0	1.0	7.0	19	20	0	1	122.0	124.0	FSIQ WAIS
Dziobek (b)	2006	0.0	0.0	1.0	1.0	2.0	1.0	1.0	1.0	7.0	17	17	0	1	113.0	115.0	FSIQ WAIS-R
Flood	2011	0.0	1.0	1.0	0.0	2.0	1.0	1.0	1.0	7.0	26	24	0	0	170.2	172.5	BPVS
Gonzalez-Gadea	2013	0.0	0.0	1.0	0.0	2.0	1.0	1.0	1.0	6.0	23	21	0	1	37.4	37.1	WAT
Grainger et al.	2014	1.0	1.0	1.0	1.0	2.0	1.0	1.0	1.0	9.0	18	18	0	1	112.33	114.94	FSIQ WASI
Heavey et al.	2000	0.0	1.0	1.0	1.0	2.0	1.0	1.0	1.0	8.0	16	15	1	0	89.63	95.53	FSIQ WAIS-R
Hill et al.	2004	0.0	1.0	0.0	0.0	0.5	0.0	1.0	1.0	3.5	15	15	0	0	91.71	116.53	
Jolliffe & Baron-Cohen	1999	0.0	1.0	1.0	1.0	2.0	1.0	0.0	1.0	7.0	34	17	0	1	106.12	106.35	FSIQ WAIS-R
Klin	2000	1.0	1.0	1.0	0.0	1.5	1.0	1.0	1.0	7.5	40	20	0	0	96.8	103.1	FSIQ mix
Kristen	2014	1.0	1.0	1.0	1.0	2.0	1.0	1.0	1.0	9.0	20	20	0	0	100.82	103.85	NVIQ CFT 20-R
Lahera	2014	0.0	0.0	0.0	1.0	1.0	0.0	1.0	0.0	3.0	22	25	0	1	NA	NA	NA
Lever & Geurts	2016	1.0	1.0	1.0	1.0	2.0	0.0	1.0	1.0	8.0	118	118	0	1	114.8	114.3	

© 2019 Rødgaard E-M et al. *JAMA Psychiatry.*

Lind et al.	2013	0.0	1.0	1.0	1.0	2.0	1.0	1.0	1.0	8.0	27	29	0	1	112.37	114.07	FSIQ WASI
Lugnegaard et al.	2013	0.0	1.0	1.0	1.0	2.0	1.0	1.0	1.0	8.0	53	50	0	0	10.4	9.9	
Marsh et al.	2011	0.5	0.0	1.0	1.0	1.5	0.0	1.0	1.0	6.0	18	19	0	0	104.4	113.4	PIQ WAIS
Martinez	2017	1.0	1.0	1.0	1.0	2.0	0.0	1.0	1.0	8.0	19	20	0	1	108.6	108.9	FSIQ WAIS III
Muller	2016	1.0	1.0	1.0	1.0	1.5	1.0	1.0	1.0	8.5	33	23	0	0	101.1	109.8	WISC-IV
Murray	2017	1.0	1.0	1.0	1.0	2.0	0.0	1.0	1.0	8.0	20	20	0	1	105.05	111.25	VIQ WAIS
Oakley	2016	0.5	1.0	1.0	1.0	2.0	1.0	1.0	1.0	8.5	19	24	0	0	109.79	108.48	WASI
Pedreno	2017	1.0	1.0	1.0	1.0	1.0	0.0	0.0	1.0	6.0	35	35	1	0	100.0	115.2	
Schaller & Rauh	2017	0.5	0.0	1.0	1.0	2.0	1.0	1.0	1.0	7.5	23	22	0	0	105.65	103.77	CFT 20-R
Schneider	2013	1.0	0.0	1.0	1.0	2.0	0.0	1.0	1.0	7.0	18	16	1	0	112.61	113.94	FSIQ WASI
Schuwerk	2015	0.0	1.0	1.0	1.0	2.0	0.0	1.0	1.0	7.0	17	17	0	0	91.4	98.3	NVIQ CFT 20-R
Segura	2015	1.0	1.0	1.0	1.0	2.0	0.0	1.0	1.0	8.0	21	10	0	0	102.0	110.0	WAIS-III
Senju	2009	0.5	1.0	1.0	0.0	1.5	1.0	1.0	1.0	7.0	19	17	0	0	115.6	115.3	
Spek	2010	1.0	1.0	1.0	1.0	2.0	1.0	1.0	1.0	9.0	61	32	0	1	112.4	115.9	FSIQ WAIS
Torralva	2013	0.0	NA	0.0	0.0	1.0	0.0	1.0	1.0	3.0	25	25	0	0	NA	NA	NA
White	2011	1.0	1.0	1.0	1.0	2.0	1.0	1.0	1.0	9.0	16	15	0	0	106.44	110.47	
White	2014	1.0	0.0	1.0	0.0	2.0	0.0	1.0	1.0	6.0	22	11	0	0	99.0	103.0	PIQ WISC-III
Wilson	2014	0.5	1.0	1.0	1.0	2.0	1.0	1.0	1.0	8.5	89	89	0	1	110.0	114.0	FSIQ WASI
Yeh	2010	0.0	0.0	1.0	1.0	2.0	0.0	1.0	1.0	6.0	22	23	0	0	94.67	93.44	FSIQ WISC
Zalla & Leboyer	2011	1.0	1.0	1.0	0.0	1.5	0.0	1.0	1.0	6.5	20	28	0	1	93.5	97.7	FSIQ WAIS-III
Zalla	2009	1.0	1.0	1.0	1.0	2.0	1.0	1.0	1.0	9.0	15	15	0	1	114.8	115.3	FSIQ WAIS-III
Zalla	2015	1.0	1.0	1.0	1.0	2.0	1.0	1.0	1.0	9.0	19	19	0	0	96.3	101.2	FSIQ WAIS III
Zalla	2016	1.0	1.0	1.0	1.0	2.0	1.0	1.0	1.0	9.0	21	21	1	0	101.71	102.76	FSIQ WAIS III
Adler et al.	2010	0.0	0.0	1.0	0.0	1.0	0.0	1.0	1.0	4.0	16	21	0	0	NA	NA	WAIS
David et al.	2008	0.0	1.0	1.0	1.0	2.0	1.0	1.0	1.0	8.0	24	24	0	0	130.1	135.7	IQ WAIS-R
Happe	1994	0.0	1.0	0.0	0.0	0.5	1.0	0.0	1.0	3.5	18	10	0	0	87.3	NA	

© 2019 Rødgaard E-M et al. *JAMA Psychiatry.*

Ponnet et al.	2004	0.0	1.0	1.0	0.0	2.0	1.0	1.0	1.0	7.0	19	19	0	0	106.58	114.5	FSIQ mix
Roeyers et al.	2001	0.0	1.0	1.0	0.0	0.0	1.0	1.0	1.0	5.0	24	24	0	0	110.3	NA	PIQ WAIS

© 2019 Rødgaard E-M et al. *JAMA Psychiatry.*

eTable 14. NOS Ratings for Cognitive Flexibility

The Newcastle-Ottawa Scale (NOS) for Assessing the Quality of Studies Included - AUTISM adaptation																	
		Selection (Tot = 4)				Comparability (Tot=2)	Outcome (Tot=3)			NOS Total	n autistic group	n control group	Autism group composition	Syn-dromic autism	IQ autistic group	IQ control group	IQ TEST
	item #	1	2	3	4	5	6	7	8								
Cognitive flexibility construct																	
Ambery et al.	2006	0.0	1.0	1.0	1.0	2.0	1.0	1.0	1.0	8.0	27	20	0	1.0	103.7	109.4	PIQ WAIS-R
Bennetto et al.	1996	0.5	0.0	1.0	1.0	2.0	1.0	1.0	1.0	7.5	19	19	0	0.0	88.9	91.7	FSIQ WISC-R
Chan et al. (a)	2011	0.0	1.0	1.0	1.0	2.0	1.0	1.0	1.0	8.0	20	20	0	1.0	89.5	101.0	FSIQ C-WISC
Geurts	2004	0.5	1.0	0.0	1.0	1.0	1.0	1.0	1.0	6.5	41	41	1	0.0	98.3	111.5	FSIQ WISC-R
Goddard	2014	0.0	1.0	1.0	1.0	2.0	1.0	1.0	1.0	8.0	63	63	0	0.0	103.6	104.8	FSIQ WASI
Goldstein et al.	2001	1.0	1.0	0.0	1.0	2.0	1.0	1.0	1.0	8.0	103	103	1	1.0	97.6	99.1	FSIQ WISC-R or WAIS-R
Griebling et al.	2010	1.0	1.0	1.0	1.0	2.0	1.0	1.0	0.0	8.0	24	38	1	1.0	104.0	104.0	FSIQ WISC-R or WAIS-R
Hill & Bird	2006	0.0	1.0	1.0	0.0	1.5	1.0	1.0	1.0	6.5	22	22	0	0.0	110.5	107.9	FSIQ WAIS-III
Kado et al.	2012	0.0	0.0	0.0	1.0	0.5	1.0	1.0	1.0	4.5	52	52	0	0.0	97.7	NA	FSIQ WISC-III
Kaland et al.	2008	1.0	0.0	1.0	0.0	2.0	1.0	1.0	1.0	7.0	13	13	0	0.0	109.0	109.6	FSIQ WISC-III
Kilincaslan	2010	0.0	1.0	1.0	1.0	2.0	1.0	1.0	1.0	8.0	21	18	0	1.0	105.5	107.3	FSIQ WISC-R
Lam & Yeung	2012	0.0	0.0	1.0	0.0	2.0	1.0	1.0	1.0	6.0	12	12	0	0.0	22.8	24.1	NVIQ CPM
Li	2014	1.0	1.0	0.0	0.0	2.0	1.0	1.0	1.0	7.0	37	31	1	0.0	109.8	113.0	NVIQ RPM
Liss et al.	2001	0.0	1.0	1.0	1.0	1.5	1.0	1.0	1.0	7.5	21	34	1	0	92.8	97.5	FSIQ WISC-R
Lopez et al.	2005	1.0	1.0	0.0	1.0	2.0	1.0	1.0	1.0	8.0	17	17	1	0	77.0	89.0	FSIQ WAIS-III
Maes et al	2011	0.0	0.0	1.0	0.0	2.0	1.0	1.0	0.0	5.0	17	19	0	0	11.0	11.0	NVIQ APM
Minshew et al.	2002	1.0	1.0	0.0	1.0	2.0	1.0	1.0	1.0	8.0	90	107	1	0	98.0	100.9	FSIQ WAIS-R

© 2019 Rødgaard E-M et al. *JAMA Psychiatry.*

Minshew et al.	1992	0.5	1.0	1.0	1.0	2.0	1.0	1.0	1.0	8.5	15	15	1	1	95.7	96.5	FSIQ WAIS-R	
Minshew et al.	1997	1.0	1.0	1.0	1.0	2.0	1.0	1.0	1.0	9.0	33	33	1	1	100.1	100.5	FSIQ WAIS-R	
Ozonoff et al.	1991	1.0	1.0	1.0	1.0	2.0	1.0	1.0	1.0	9.0	23	20	0	0	89.5	91.3	FSIQ WISC-R or WAIS-R	
Ozonoff & McEvoy	1994	1.0	0.0	1.0	1.0	2.0	1.0	1.0	0.0	7.0	17	17	0	0	89.1	95.6	FSIQ WISC-R or WAIS-R	
Ozonoff study 1	1995	1.0	0.0	1.0	1.0	2.0	1.0	1.0	1.0	8.0	17	17	0	1	89.1	95.6	FSIQ WISC-R	
Ozonoff study 2	1995	0.0	1.0	1.0	1.0	1.5	1.0	1.0	1.0	7.5	10	11	1	0	98.1	99.1	FSIQ WISC-III	
Ozonoff study 3	1995	0.0	1.0	1.0	1.0	1.5	1.0	1.0	1.0	7.5	24	24	1	0	97.4	101.9	FSIQ WISC-III	
Ozonoff & Jensen	1999	1.0	1.0	0.0	1.0	0.5	1.0	1.0	1.0	6.5	40	29	1	0	95.2	107.8	FSIQ WISC-III	
Pascualvaca et al.	1998	1.0	1.0	1.0	1.0	1.5	1.0	1.0	1.0	8.5	23	23	1	1	77.6	110.2	FSIQ WISC-III	
Prior	1990	0.0	0.0	0.0	1.0	1.0	1.0	1.0	1.0	5.0	12	12	1	1	88.0	100.0	LIPS	
Robinson	2009	0.0	0.0	1.0	1.0	2.0	1.0	1.0	1.0	7.0	54	54	0	0	103.5	104.8	FSIQ WASI	
Rumsey	1985	0.0	1.0	0.0	1.0	2.0	1.0	1.0	1.0	7.0	9	10	1	1	104.0	113.0	FSIQ WAIS	
Sawa	2013	0.0	1.0	1.0	1.0	2.0	1.0	1.0	1.0	8.0	19	19.0	0	0	96.0	97.3	FSIQ WISC-III	
Schneider & Asarnow test 1	1987	0.0	1.0	1.0	1.0	0.5	1.0	1.0	0.0	5.5	13.0	28.0	1	1	85.7	NA	FSIQ WISC-R	
Shu	2001	0.0	1.0	0.0	0.0	1.0	1.0	1.0	1.0	5.0	26.0	52.0	1	1	80.0	NA	FSIQ WISC	
Sumiyoshi	2011	0.5	1.0	0.0	1.0	1.5	1.0	1.0	1.0	7.0	22.0	15.0	0	1	94.1	99.7	FSIQ WISC-III or WAIS-R	
Szatmari et al.	1990	0.0	NA	0.0	1.0	0.0	1.0	1.0	1.0	4.0	17.0	36.0	1	0	82.2	101.5	FSIQ WISC-R or WAIS-R	
Tsuchiya	2005	0.0	1.0	0.0	1.0	0.5	1.0	1.0	1.0	5.5	17.0	25.0	1	0	92.3	NA	FSIQ WISC-R or WISC-III	
van Eylen	2011	0.0	1.0	1.0	1.0	2.0	1.0	1.0	1.0	8.0	40.0	40.0	0	1	105.5	106.8	FSIQ WISC-III or WAIS-III	
van Eylen	2015	0.0	0.0	1.0	1.0	2.0	1.0	1.0	1.0	7.0	50.0	50.0	0	1	104.3	107.7	FSIQ WISC-III or WAIS-III	
Vanegas	2015	0.0	1.0	0.0	1.0	0.0	1.0	1.0	1.0	5.0	13.0	25.0	1	0	95.5	110.1	NVIQ RPM	
Verte	2006	1.0	1.0	0.0	1.0	1.0	1.0	1.0	1.0	7.0	50.0	47.0	1	0	98.2	112.1	FSIQ WISC-R	
Voelbel	2006	1.0	1.0	0.0	1.0	1.0	1.0	1.0	1.0	7.0	38.0	13.0	0	1	99.4	115.2	FSIQ WISC-III	

© 2019 Rødgaard E-M et al. *JAMA Psychiatry.*

Mean of Willam & Jarrold and Williams et al. (2013)	2013	0.0	1.0	1.0	1.0	1.5	1.0	1.0	1.0	7.5	21.0	22.0	0	0	110.2	107.2	PIQ WASI	
Winsler	2007	0.0	NA	0.0	0.0	0.5	1.0	1.0	1.0	3.5	33.0	28.0	0	0	NA	NA	NA	
Yang et al.	2009	0.0	1.0	0.0	1.0	0.5	1.0	1.0	1.0	5.5	20.0	30.0	0	0	96.7	118.2	NVIQ GNIT	
Yasuda et al.	2014	1.0	1.0	1.0	1.0	2.0	1.0	1.0	1.0	9.0	33.0	33.0	0	0	103.0	103.7	FSIQ WAIS-III	
Narzisi	2013	0	1	0	0	1	1	1	1	5.0	22	44	0	1	99.09	NA	FSIQ WISC-III	
Perez	2009	0	1	1	1	1.5	1	1	1	7.5	15	16	0	0	111.2	123	FSIQ WASI	
Russel-smith	2014	0.0	1	0.0	1.0	2.0	1.0	1.0	1.0	7.0	17.0	18.0	1	0	101.6	102.9	WISC-IV	
Semrud-Clikeman	2010	0.5	1	1	1	1.5	1	1	1	8.0	15	32	0	1	100.8	109.4	FSIQ WASI	
Semrud-Clikeman	2014	1	1	0	1	1	1	1	1	7.0	36	38	0	0	102.9	113	FSIQ WASI	
Czermainski	2014	0	NA	1	0	2	1	1	1	6.0	11	19	0	0	NA	NA	RCPM-Special scale	

© 2019 Rødgaard E-M et al. *JAMA Psychiatry.*

eTable 15. NOS Ratings for Planning

The Newcastle-Ottawa Scale (NOS) for Assessing the Quality of Studies Included - AUTISM adaptation																	
		**Selection (Tot = 4)**				**Comparability (Tot=2)**	**Outcome (Tot=3)**			NOS Total	n autistic group	n control group	Autism group composition	Syn-dromic autism	IQ autistic group	IQ control group	IQ TEST
	item #	**1**	**2**	**3**	**4**	**5**	**6**	**7**	**8**								
Theory of mind (meta-analysis from Leppanen et al., 2018)																	
Corbett	2009	1.0	1.0	0.0	1.0	1.0	1.0	1.0	1.0	7.0	18	18	0	1.0	94.2	112.22	WASI
Goldberg	2005	1.0	1.0	0.0	1.0	0.5	1.0	1.0	1.0	6.5	17	32	1	1	96.5	112.6	WISC-3
Happe	2006	0.0	1.0	1.0	1.0	2.0	1.0	1.0	1.0	8.0	32	32	0	0	99.7	106.8	WISC-3
Hughes	1994	0.0	1.0	0.0	1.0	0.5	1.0	1.0	1.0	5.5	35	47	1	0	NA	NA	NA
Kaufmann	2013	1.0	1.0	1.0	1.0	2.0	1.0	1.0	1.0	9.0	10	10	0	0	102.3	109.5	WISC-3
Landa & Goldberg	2005	1.0	1.0	1.0	1.0	2.0	1.0	1.0	1.0	9.0	19	19	1	1	109.7	113.4	WISC-3
Ozonoff	2004	1.0	1.0	1.0	1.0	2.0	1.0	1.0	1.0	9.0	79	70	1	0	106.3	106.0	WISC-3
Sachse	2013	1.0	1.0	1.0	1.0	2.0	1.0	1.0	1.0	9.0	30	28	0	1	105.3	109.3	SPM
Sinzig	2008	1.0	1.0	0.0	1.0	0.5	1.0	1.0	1.0	6.5	20	20	0	1	112.0	113.0	CFIT
Bolte	2011	1.0	0.0	1.0	1.0	1.5	1.0	1.0	1.0	7.5	56	78	0	1	99.2	103.5	SPM/WISC-3(performance IQ)
Griebling	2010	1.0	NA	1.0	1.0	2.0	1.0	1.0	1.0	8.0	38	40	1	1	NA	NA	WAIS
Hanson & Atance	2014	0.0	1.0	1.0	0.0	1.0	1.0	1.0	1.0	6.0	25	25	0	0	85.7	109.1	WIPPSI-3
Keary	2009	1.0	1.0	1.0	1.0	2.0	1.0	1.0	1.0	9.0	32	34	1	1	102.9	104.0	WISC
Losh	2009	0.5	1.0	0.0	1.0	1.0	1.0	1.0	1.0	6.5	36	41	1	0	101.2	108.3	WISC
Medeiros & Winsler	2014	0.0	NA	0.0	1.0	0.5	1.0	1.0	1.0	4.5	32	26	0	0	NA	NA	
Ozonoff & Jensen	1999	0.5	1.0	0.0	1.0	0.5	1.0	1.0	1.0	6.0	40	29	NA	0	95.2	107.8	WISC-3
Williams	2014	1.0	1.0	1.0	1.0	2.0	1.0	1.0	1.0	9.0	65	65	1	1	98.8	102.1	N/A
Geurts	2004	0.5	1.0	0.0	1.0	1.0	1.0	1.0	1.0	6.5	42	41	1	0	98.3	111.5	WISC
Geurts & Vissers	2012	0.0	1.0	1.0	1.0	2.0	1.0	1.0	1.0	8.0	23	23	0	0	109.5	109.8	DART-IQ
Kimhi	2014	1.0	1.0	1.0	0.0	2.0	1.0	1.0	1.0	8.0	29	30	0	0	103.5	107.6	WISC/WIPPSI

© 2019 Rødgaard E-M et al. *JAMA Psychiatry.*

Limoges	2013	1.0	1.0	1.0	1.0	2.0	1.0	1.0	1.0	9.0	17	14	0	0	104.1	112.3	WISC-3	
Panerai	2014	0.0	1.0	1.0	0.0	2.0	1.0	1.0	1.0	7.0	11	9	0	0	25.0	23.0	RAVEN	
Pellicano	2010	1.0	1.0	0.0	1.0	2.0	1.0	1.0	0.0	7.0	37	31	0	0	113.3	115.6	Leiter-R	
Robinson	2009	1.0	0.0	1.0	1.0	2.0	1.0	1.0	1.0	8.0	54.0	54.0	NA	0.0	103.5	104.8	WASI	
Schurink	2012	0.0	0.0	0.0	1.0	1.0	1.0	1.0	1.0	5.0	28.0	28.0	0	0.0	81.4	NA	NA	
Unterrainer	2015	1.0	1.0	1.0	1.0	2.0	1.0	1.0	1.0	9.0	18.0	42.0	0.0	0.0	97.1	97.6	SPM	
Verte	2005	1.0	1.0	0.0	1.0	1.0	1.0	1.0	1.0	7.0	61.0	47.0	1.0	0.0	99.2	112.1	WISC-R	
Verte	2006	1.0	1.0	0.0	1.0	1.0	1.0	1.0	1.0	7.0	112.0	47.0	0	0.0	100.6	112.1		
Wallace	2009	1.0	1.0	1.0	1.0	2.0	1.0	1.0	1.0	9.0	28.0	25.0	0	1.0	110.3	113.8	WISC/WAIS	
Williams & Jarrold	2013	0.0	1.0	1.0	1.0	1.5	1.0	1.0	1.0	7.5	21.0	22.0	0	0.0	110.2	107.18	WISC	
Williams	2012	1.0	1.0	1.0	1.0	1.5	1.0	1.0	1.0	8.5	15.0	16.0	0	0.0	114.0	116.71	WAIS	
Zinke	2010	1.0	1.0	0.0	0.0	1.0	1.0	1.0	1.0	6.0	15.0	17.0	0	1.0	96.4	NA	WISC	
Boucher	2005	0.0	1.0	1.0	1.0	2.0	1.0	1.0	1.0	8.0	10.0	10.0	1	0.0	105.5	104.4	WAIS	
Bramham	2009	1	1	0	1	2	1	1	1	8.0	45	31	0	1	107	109.84	WAIS	
Hill & Bird	2006	0	1	1	0	2	1	1	1	7.0	22	22	0	0	110.5	107.91	WAIS	
Rajendran	2005	0	1	1	0	2	1	1	1	7.0	12	12	0	0	102	109	WISC/WAIS	
White	2009	1	0	1	1	2	1	1	1	8.0	45	27	0	0	98	103	WISC-PIQ	
Joseph	2005	1	1	1	1	2	1	1	1	9.0	37	31	1	1	91	91	DAS	
McCrimmon	2012	0	0	0	1	2	1	1	1	6.0	33	33	0	0	113.18	110.06	WAIS	
Planche & Lemonnier	2012	1	1	1	1	2	1	1	1	9.0	30	15	0	0	101.8	106.2	WISC	
Semrud-Clikeman	2010	1	1	0	1	1	1	1	1	7.0	32	15	0	0	100.8	109.4	WASI	
Van Eylen	2015	0	0	1	1	2	1	1	1	7.0	50	50	0	0	104.32	107.72	WISC/WAIS	
Goddard	2014	0	1	1	1	2	1	1	1	8.0	63	63	0	0	103.6	104.76	WISC	
Low	2009	0	NA	0	0	1.5	1	1	1	4.5	27	27	0	0	NA	NA	NA	
Pellicano	2006	1	1	1	1	2	1	1	1	9.0	40	40	0	1	113.58	112.52	Leiter-R	
Pellicano	2007	1	1	1	1	2	1	1	1	9.0	30	40	1	0	113.87	112.52	Leiter-R	

© 2019 Rødgaard E-M et al. *JAMA Psychiatry.*

eTable 16. NOS Ratings for Inhibition

The Newcastle-Ottawa Scale (NOS) for Assessing the Quality of Studies Included - AUTISM adaptation																	
		**Selection (Tot = 4)**				**Comparability (Tot=2)**	**Outcome (Tot=3)**			NOS Total	n autistic group	n control group	Autism group composition	Syndromic autism	IQ autistic group	IQ contro l group	IQ TEST
	item #	**1**	**2**	**3**	**4**	**5**	**6**	**7**	**8**								
Inhibition																	
Adamo	2014	1	1	1	1	1.5	1	1	1	8.5	46	36	0	1	109	112	FSIQ WASI
Adams & Jarrold	2009	0	NA	1	1	1	1	1	1	6.0	24	24	0	1	27.71	26.25	RCPM **RAW SCORE** (MAX 36)
Adams & Jarrold	2012	0	NA	1	1	1	1	1	1	6.0	15	15	0	1	25.86	24.53	RCPM **RAW SCORE** (MAX 36)
Christ	2007	0	1	0	1	0	1	1	1	5.0	18	25	0	0	88.4	107.7	FSIQ WASI
Christ	2011	1	1	1	1	0.5	1	1	0	6.5	28	49	0	0	99.7	107.6	Leiter
Dichter & Belger	2008	0.5	1	1	1	1.5	1	1	1	8.0	12	22	0	1	106.9	109.8	FSIQ WASI
Geurts	2008	0	1	1	1	2	1	1	1	8.0	22	33	0	1	102.7	103.3	FSIQ WISC-III Dutch short version
Larson	2012	0.5	1	1	1	2	1	1	1	8.5	28	36	0	0	105	109	FSIQ WASI
South	2010	0.5	1	1	1	2	1	1	1	8.5	24	21	0	0	109.71	112.05	FSIQ WASI
Brandimonte	2011	0.5	0	1	0	2	1	1	1	6.5	10	10	1	1	87.03	89.03	FSIQ WISC-III Italian version
Geurts	2009	0	1	1	1	2	1	1	1	8.0	18	22	0	0	108	103.2	FSIQ WISC-III Dutch short version
Happé	2006	0	1	1	1	2	1	1	1	8.0	32	32	0	0	99.7	106.8	FSIQ WISC-III (short version for some ctrl children)
Langen	2012	1	1	1	1	1.5	1	1	1	8.5	21	22	1	1	107.45	109.82	FSIQ WASI
Lee	2009	1	1	1	1	2	1	1	1	9.0	12	12	0	0	113.33	114.92	FSIQ test not mentionned
Ozonoff	1994	0	1	1	1	2	1	1	1	8.0	14	14	0	0	101.9	100.4	FSIQ WISC-III
Sanderson & Allen	2013	1	NA	1	1	1	1	1	1	7.0	31	28	0	0	28.1	29.46	RCPM **RAW SCORE** (MAX 36)
Schmitz	2006	0	1	1	1	2	1	1	1	8.0	10	11	0	1	105	106	FSIQ WAIS-R short form
Sinzig	2008	0.5	1	1	1	2	1	1	1	8.5	20	20	0	1	112	113	IQ Culture Fair Intelligence Test
Sinzig	2014	1	1	1	1	0	1	1	1	7.0	26	29	0	1	90.5	107.4	IQ Kaufmann-Assessment Battery for Children

© 2019 Rødgaard E-M et al. *JAMA Psychiatry.*

Xiao	2012	1	0	1	1	2	1	1	1	8.0	19	16	1	1	99.26	105.63	FSIQ WISC-II
van Eylen	2015	0	0	1	1	2	1	1	1	7.0	50	50	0	1	104.32	107.72	WISC-III-NL or WAIS-III-NL
Chan (b)	2014	1	1	1	1	2	1	1	1	9.0	20	20	0	0	101.4	110.7	TONI-III (deviation quotient)
Samyn	2015	0	1	0	0	0.5	1	1	1	4.5	31	148	0	0	101.16	107.21	FSIQ WISC-III short version
Pankert	2014	0	1	1	0	2	1	1	1	7.0	17	17	0	0	109.3	109.2	FSIQ WISC-III short version
Kretschmer	2014	1	NA	1	1	0	1	1	1	6.0	21	21	0	1	NA	NA	Children just had completed WISC-IV vocabulary and matrices
Vara	2014	1	1	1	1	1	1	1	1	8.0	15	15	0	1	103.8	112.4	WASI vocabulary and matrices subtests
Ambrosino	2014	0.5	1	1	1	2	1	1	1	8.5	19	19	0	1	112.2	120.2	FSIQ WISC-III
Chan (b)	2011	0.5	1	1	1	2	1	1	1	8.5	20	20	1		101.4	110.7	TONI-III (deviation quotient)
Chien	2014	1	1	1	1	0	1	1	1	7.0	215	226	1	0	89.49	111.95	FSIQ WISC-III
Kilincaslan	2010	0	1	1	1	2	1	1	1	8.0	21	18	0	0	105.52	107.27	FSIQ WISC-R
Tye	2014	1	1	1	1	1	1	1	0	7.0	19	26	0	0	115.68	120.04	FSIQ WASI
Goddard	2014	0	1	1	1	2	1	1	1	8.0	63	63	0	0	103.6	104.76	FSIQ WASI
Robinson	2009	0	0	1	1	1	1	1	1	6.0	54	54	0	0	103.53	104.8	WASI vocabulary and matrices subtests
Mahone	2006	0.5	1	1	1	0	1	0	1	5.5	24	60	0	1	99.1	118.2	FSIQ WISC-III
Pellicano	2006	1	1	1	1	2	1	1	1	9.0	40	40	0	1	113.58	112.52	NVIQ Leiter
Bishop & Norbury	2005	0.5	1	1	0	1	1	1	1	6.5	14	18	0	0	98.94	105	Raven Matrices scale score
Geurts	2004	0.5	1	1	1	1	1	1	0	6.5	41	41	0	0	98.3	111.5	FSIQ WISC-R or WISC-R short version
Verte	2006	0.5	1	1	1	1	1	1	1	7.5	112	47	0	0	100.5	112.1	FSIQ WISC-R
Lemon	2011	0	1	1	0	2	1	1	1	7.0	23	22	0	0	94.49	107.5	FSIQ Wechsler version not mentionned
Ozonoff & Strayer	1997	0	1	1	1	2	1	1	1	8.0	13	13	1	0	101	100.1	FSIQ WISC-III
Andersen	2015	0	1	1	1	2	1	1	1	8.0	34	45	0	1	99.9	104.5	FSIQ WASI
Corbett	2009	0.5	1	1	1	1.5	1	1	1	8.0	18	18	0	1	94.17	112.22	FSIQ WASI
Semrud-Clikeman	2010	0.5	1	1	1	1.5	1	1	1	8.0	15	32	0	1	100.8	109.4	FSIQ WASI
Barron-Linnankoski	2015	1	1	0	1	1	1	1	1	7.0	30	60	0	1	107.2	NA	FSIQ WISC-III
Narzisi	2013	0	1	0	0	1	1	1	1	5.0	22	44	0	1	99.09	NA	FSIQ WISC-III

© 2019 Rødgaard E-M et al. *JAMA Psychiatry.*

Terrett	2013	0	1	1	0	1.5	1	1	1	6.5	30	30	0	0	115.63	116.57	FSIQ WASI
Chan (a)	2011	0	1	1	1	2	1	1	1	8.0	20	20	0	0	89.5	101	FSIQ WISC chinese short version
Perez	2009	0	1	1	1	1.5	1	1	1	7.5	15	16	0	0	111.2	123	FSIQ WASI
Czemainski	2014	0	NA	1	0	2	1	1	1	6.0	11	19	0	0	NA	NA	RCPM-Special scale
Goldberg	2005	0.5	1	1	1	0.5	1	1	1	7.0	17	32	1	1	96.5	112.6	FSIQ WISC-R or WISC-III
Voelbel	2006	0	1	1	1	1	1	1	1	7.0	38	13	0	0	99.37	115.15	FSIQ WISC-III
Weissman	2010	0.5	0	0	1	0	1	1	1	4.5	48	26	0	0	49.9	58.36	FSIQ WISC-III
Maister (a)	2013	0.5	1	1	1	1.5	1	1	1	8.0	14	14	0	0	109.5	120.6	British Picture Vocabulary Scale Standardized
Yasumura	2014	0	NA	1	1	1.5	1	1	1	6.5	11	15	0	1	30.45	29.47	RCPM RAW SCORE (MAX 36)
Yoran-Hegesh	2009	0	NA	0	0	1	1	1	1	4.0	23	43	0	0	NA	NA	NA
Jahromi	2013	0.5	NA	0	0	1.5	1	1	1	5.0	20	20	1	0	NA	NA	NA
Johnston	2011	1	1	1	0	2	1	1	1	8.0	24	14	0	0	102.7	108	VIQ WAIS-III or WASI
Ozonoff & Jensen	1999	0.5	1	1	1	0.5	1	1	1	7.0	40	29	1	0	95.2	107.8	FSIQ WISC-III
Russell	1999	0	1	1	0	0.5	1	1	1	5.5	19	19	1	0	88	87.89	VMA British Picture Voc Scale
Yerys (a)	2009	0.5	1	1	1	2	1	1	1	8.5	28	21	0	1	117.39	116.24	FSIQ WISC-III, WISC-IV, WASI
Zandt	2009	0	1	1	1	1.5	1	1	1	7.5	19	18	0	1	95.38	102.72	PIQ WISC-III
Henry	2014	0	1	1	1	2	1	1	1	8.0	30	30	0	1	112.93	115.3	FSIQ WASI
Ames & Jarrold	2007	0	NA	1	1	0.5	1	1	1	5.5	15	15	0	1	22.87	16.53	RCPM RAW SCORE (MAX 36)

© 2019 Rødgaard E-M et al. *JAMA Psychiatry.*

eTable 17. NOS Ratings for P3b Amplitude

The Newcastle-Ottawa Scale (NOS) for Assessing the Quality of Studies Included - AUTISM adaptation																	
		Selection (Tot = 4)				Comparability (Tot=2)	Outcome (Tot=3)			NOS Total	n autistic group	n control group	Autism group composition	Syndromic autism	IQ autistic group	IQ control group	IQ TEST
	item #	1	2	3	4	5	6	7	8								
P3b from Cui et al. (2017)																	
Novick et al.	1980	0	0	0	1.0	0.5	1.0	1.0	1.0	4.5	5	5	1	0	NA	NA	NA
Courchesne et al. (1984)	1984	0.0	0.0	0.0	0.0	1.0	1.0	1.0	1.0	4.0	7	7	1	0	NA	NA	NA
Oades et al.	1988	0.0	1.0	1.0	0.0	1.0	1.0	1.0	0.0	5.0	7	9	1	0	90.0	123.0	Leiter scale
Courchesne et al. (1989)	1989	0.0	0.0	0.0	0.0	0.5	1.0	1.0	1.0	3.5	11	16	1	0	90.0	110.0	WISC (performance IQ)
Ciesielski et al.	1990	0.0	1.0	1.0	0.0	1.0	1.0	1.0	0.0	5.0	10	13	1	0	99.0	107.0	WAIS (performance IQ)
Verbaten et al.	1991	0.0	1.0	0.0	1.0	1.0	1.0	1.0	1.0	6.0	20	20	1	0	92.0	114.0	NA
Erwin et al.	1991	0.0	1.0	0.0	0.0	0.5	1.0	1.0	1.0	4.5	11	14	1	0	97.0	NA	NA
Lincoln et al.	1993	0.0	0.0	0.0	1.0	0.5	1.0	1.0	0.0	3.5	8	10	1	0	71.1	108.6	WISC
Senju et al.	2005	0.0	0.0	0.0	0.0	1.0	1.0	1.0	1.0	4.0	13	15	1	0	NA	NA	RCPM
Salmond et al.	2007	0.0	1.0	1.0	1.0	1.0	1.0	1.0	1.0	7.0	26	19	0	1	89.0	107.5	WISC
Kohls et al.	2011	1.0	1.0	1.0	1.0	2.0	1.0	1.0	1.0	9.0	16	20	0	0	108.6	109.9	WISC
Clery et al. (2)	2013	1.0	1.0	0.0	1.0	1.0	1.0	1.0	1.0	7.0	12	12	0	0	92.0	NA	nv(DQ)
Andersson et al. (2013)	2013	0.0	0.0	0.0	1.0	2.0	1.0	1.0	1.0	6.0	11	12	0	0	99.2	99.2	WISC (Performance IQ)
Tye et al.	2014	1.0	1.0	0.0	1.0	2.0	1.0	1.0	1.0	8.0	19	26	0	0	115.7	120.0	WASI

© 2019 Rødgaard E-M et al. *JAMA Psychiatry.*

eTable 18. NOS Ratings for Brain Size

The Newcastle-Ottawa Scale (NOS) for Assessing the Quality of Studies Included - AUTISM adaptation																	
		**Selection (Tot = 4)**				**Comparability (Tot=2)**	**Outcome (Tot=3)**			NOS Total	n autistic group	n control group	Autism group composition	Syn-dromic autism	IQ autistic group	IQ control group	IQ TEST
	item #	**1**	**2**	**3**	**4**	**5**	**6**	**7**	**8**								
Brain size (meta-analysis from Sacco et al., 2015)																	
Piven	1995	0.5	1.0	1.0	1.0	1.0	1.0	1.0	1.0	7.5	22	20	1	1	90.8	103.4	Autistic group: PIQ WAIS-R, WISC-R or Leiter; Control group: PIQ WAIS-R or WISC-III
Aylward	1999	1.0	1.0	1.0	1.0	2.0	1.0	1.0	1.0	9.0	14	14	1	1	106.4	108.5	FSIQ, test not mentionned
Haznedar	2000	0.0	1.0	0.0	1.0	1.0	1.0	1.0	1.0	6.0	17	17	0	1	NA	NA	FSIQ, test not mentionned
Hardan	2000	1.0	1.0	1.0	1.0	1.0	1.0	1.0	0.0	7.0	16	19	1	1	100.4	100.5	FSIQ WAIS-R or WISC-R
Courchesne (a)	2001	1.0	NA	0.0	1.0	0.5	1.0	0.0	1.0	4.5	30	12	1	0	NA	NA	NVIQ WISC-III, WISC-R, Leiter or Stanford Binet
Courchesne (b)	2001	1.0	NA	0.0	1.0	0.5	1.0	0.0	1.0	4.5	15	14	1	0	NA	NA	NVIQ WISC-III, WISC-R, Leiter or Stanford Binet
Courchesne (c)	2001	1.0	NA	0.0	1.0	0.5	1.0	0.0	1.0	4.5	10	14	1	0	NA	NA	NVIQ WISC-III, WISC-R, Leiter or Stanford Binet

© 2019 Rødgaard E-M et al. *JAMA Psychiatry.*

Pierce & Courchesne	2001	0.5	1.0	0.0	0.0	0.5	1.0	1.0	1.0	5.0	14	14	1	0	84.4	110.0	Autistic group: IQ Leiter (n=11); Bayley (n=1); Abstract Reasoning Standford Binet (n=2); Control group: FSIQ Standford Binet *or* Wechsler
Aylward (a)	2002	1.0	1.0	1.0	1.0	2.0	1.0	1.0	1.0	9.0	23	28	1	1	102.7	107.0	NA
Aylward (b)	2002	1.0	1.0	1.0	1.0	2.0	1.0	1.0	1.0	9.0	20	27	1	1	102.7	107.0	NA
Aylward (c)	2002	1.0	1.0	1.0	1.0	2.0	1.0	1.0	1.0	9.0	24	28	1	1	102.7	107.0	NA
Carper (a)	2002	1.0	NA	0.0	1.0	0.5	1.0	0.0	1.0	4.5	12	8	1	0	NA	NA	Autistic group: Leiter, Standford-Binet, WISC-III, PPVT-R; Control group: PPVT-R, Standford-Binet, WISC-III
Carper (b)	2002	1.0	NA	0.0	1.0	0.5	1.0	0.0	1.0	4.5	19	17	1	0	NA	NA	
Carper (c)	2002	1.0	NA	0.0	1.0	0.5	1.0	0.0	1.0	4.5	7	14	1	0	NA	NA	
Sparks	2002	1.0	NA	0.0	1.0	0.5	1.0	0.0	1.0	4.5	45	26	0	1	NA	NA	NA
Rojas	2002	0.5	1.0	1.0	1.0	1.0	1.0	1.0	1.0	7.5	15	15	1	0	94.9	124.8	FSIQ, test not mentionned
McAlonan	2002	1.0	1.0	0.0	1.0	1.0	1.0	1.0	1.0	7.0	21	24	0	1	96.0	114.0	WAIS-R short form
Herbert	2003	0.0	NA	0.0	1.0	0.5	1.0	1.0	1.0	4.5	17	15	1	0	NA	NA	PIQ, test not mentionned
Tsatsanis	2003	1.0	1.0	1.0	1.0	2.0	1.0	1.0	1.0	9.0	12	2	1	1	106.4	108.8	FSIQ, WISC-III or WAIS-R
Hardan	2003	1.0	1.0	1.0	1.0	2.0	1.0	1.0	1.0	9.0	40	41	1	1	103.1	104.2	FSIQ, WISC-R or WAIS-R
Kates	2004	0.5	NA	0.0	1.0	1.0	1.0	1.0	0.0	4.5	9	16	1	1	NA	NA	NA

© 2019 Rødgaard E-M et al. *JAMA Psychiatry.*

Palmen	2004	0.5	1.0	1.0	1.0	1.0	1.0	1.0	1.0	7.5	21	21	0	0	114.9	112.6	FSIQ, WAIS-R
Akshoomoff (a)	2004	1.0	NA	0.0	1.0	0.0	1.0	0.0	1.0	4.0	30	15	0	1	NA	108.1	NVIQ, Stanford-Binet, WAIS-III, MSEL,Leiter, PPVT-R,Differential Abilities Scale
Akshoomoff (b)	2004	1.0	NA	0.0	1.0	0.0	1.0	0.0	1.0	4.0	12	15	1	1	NA	108.1	NVIQ, Stanford-Binet, WAIS-III, MSEL,Leiter, PPVT-R,Differential Abilities Scale
Akshoomoff (c)	2004	1.0	NA	0.0	1.0	0.0	1.0	0.0	1.0	4.0	10	15	0	1	NA	108.1	NVIQ, Stanford-Binet, WAIS-III, MSEL,Leiter, PPVT-R,Differential Abilities Scale
Schumann (a)	2004	0.5	0.0	1.0	1.0	1.0	1.0	0.0	1.0	5.5	18	22	0	1	56.0	115.0	FSIQ Leiter, WAIS ou WASI
Schumann (b)	2004	0.5	1.0	1.0	1.0	1.0	1.0	0.0	1.0	6.5	21	22	1	1	91.0	115.0	FSIQ WAIS ou WASI
Schumann (c)	2004	0.5	1.0	1.0	1.0	2.0	1.0	0.0	1.0	7.5	24	22	0	1	106.0	115.0	FSIQ WAIS ou WASI
Palmen	2005	0.5	1.0	1.0	1.0	2.0	1.0	1.0	1.0	8.5	21	21	1	0	106.5	102.5	FSIQ WISC-R
Vidal	2006	1.0	1.0	1.0	1.0	2.0	1.0	0.0	1.0	8.0	24	26	1	1	95.9	104.8	FSIQ WISC-III or Leiter
Mostofsky	2007	0.5	1.0	1.0	1.0	0.5	1.0	1.0	1.0	7.0	20	36	1	1	104.1	120.2	FSIQ WISC-III or WISC-IV
Girgis	2007	1.0	1.0	0.0	1.0	1.0	1.0	1.0	0.0	6.0	11	18	1	1	93.1	115.4	FSIQ WISC-III

© 2019 Rødgaard E-M et al. *JAMA Psychiatry.*

Bloss (a)	2007	1.0	1.0	0.0	1.0	0.0	1.0	0.0	1.0	5.0	9	14	1	1	82.8	118.7	NVIQ Differential Abilities Scale, WISC-III, Standford-Binet (or Leiter for autistic group only)
Bloss (b)	2007	1.0	1.0	0.0	1.0	0.0	1.0	0.0	1.0	5.0	27	13	1	1	82.5	109.8	NVIQ Differential Abilities Scale, WISC-III, Standford-Binet (or Leiter for autistic group only)
Tate	2007	0.5	1.0	1.0	1.0	2.0	1.0	0.0	1.0	7.5	34	26	1	1	101.7	107.0	PIQ WISC-III or WAIS-III
Hardan	2008	1.0	1.0	1.0	1.0	2.0	1.0	0.0	1.0	8.0	12	12	0	1	109.5	107.4	FSIQ WAIS-R or WISC-R
Cleavinger	2008	0.5	1.0	1.0	1.0	2.0	1.0	0.0	1.0	7.5	28	16	1	1	98.6	102.0	PIQ WISC-III or WAIS-III
Hardan	2009	1.0	1.0	1.0	1.0	1.0	1.0	1.0	0.0	7.0	18	16	1	0	93.9	113.3	FSIQ WISC-III
Hallahan (a)	2009	0.0	1.0	0.0	1.0	1.0	1.0	1.0	1.0	6.0	114	60	0	1	98.0	114.0	FSIQ WAIS-R
Hallahan (b)	2009	0.0	1.0	0.0	1.0	1.0	1.0	1.0	1.0	6.0	80	60	0	1	102	114.0	FSIQ WAIS-R
Hallahan (c)	2009	0.0	1.0	0.0	1.0	1.0	1.0	1.0	1.0	6.0	28	60	0	1	85.0	114.0	FSIQ WAIS-R
Hallahan (d)	2009	0.0	1.0	0.0	1.0	1.0	1.0	1.0	1.0	6.0	6	60	0	1	83.0	114.0	FSIQ WAIS-R
Freitag	2009	0.5	1.0	1.0	1.0	2.0	1.0	1.0	1.0	8.5	15	15	0	1	101.2	112.1	FSIQ WAIS-III German version
Scott	2009	0.5	1.0	1.0	1.0	1.0	1.0	0.0	1.0	6.5	48	14	0	1	79.0	113.0	FSIQ WISC, WASI or Leiter
Tamura (a)	2010	0.5	1.0	1.0	1.0	2.0	1.0	1.0	1.0	8.5	12	16	1	1	83.9	95.1	FSIQ WISC-III or WAIS-R

© 2019 Rødgaard E-M et al. *JAMA Psychiatry.*

Tamura (b)	2010	0.5	1.0	1.0	1.0	2.0	1.0	1.0	1.0	8.5	15	16	0	1	97.3	95.1	FSIQ WISC-III or WAIS-R
Tamura (c)	2010	0.5	1.0	1.0	1.0	2.0	1.0	1.0	1.0	8.5	11	16	0	1	86.4	95.1	FSIQ WISC-III or WAIS-R
Jou	2010	1.0	1.0	1.0	1.0	2.0	1.0	1.0	1.0	9.0	18	19	1	1	103.6	103.9	FSIQ Wechsler age appropriate version
Schumann	2010	0.5	1.0	0.0	0.0	0.0	1.0	1.0	1.0	4.5	41	44	0	0	57.0	112.0	FSIQ WPPSI-III
Jou (a)	2010	1.0	1.0	1.0	1.0	2.0	1.0	1.0	1.0	9.0	6	8	1	1	110.0	114.7	FSIQ Wechsler age appropriate version
Jou (b)	2010	1.0	1.0	1.0	1.0	2.0	1.0	1.0	1.0	9.0	9	8	0	1	112.4	114.7	FSIQ Wechsler age appropriate version
Bigler	2010	0.5	1.0	1.0	1.0	2.0	1.0	0.0	1.0	7.5	42	59	1	1	100.3	105.5	FSIQ WISC-III or WAIS-III
Griebling	2010	1.0	1.0	1.0	1.0	2.0	1.0	1.0	1.0	9.0	33	37	1	1	104.0	104.0	FSIQ WISC-R or WAIS-R
Tepest	2010	0.0	1.0	1.0	1.0	2.0	1.0	1.0	1.0	8.0	29	29	0	0	125.2	135.3	FSQI WAIS
Cheung	2011	0.0	1.0	1.0	1.0	2.0	1.0	1.0	1.0	8.0	36	55	0	1	112.0	117.0	VIQ WISC
Hong	2011	1.0	1.0	1.0	1.0	2.0	1.0	1.0	1.0	9.0	18	16	1	1	105.2	106.1	FSIQ WISC-II Chinese version
Calderoni	2012	1.0	1.0	1.0	1.0	2.0	1.0	1.0	1.0	9.0	38	38	0	1	72.0	73.0	Leiter, Griffiths Mental Dev Scale, WPPSI Italian version, WISC-R Italian version
Stamova	2013	1.0	1.0	1.0	1.0	1.0	1.0	0.0	1.0	7.0	30	20	0	0	66.7	103.6	NVQ Mullen?
Greimel	2013	1.0	1.0	1.0	1.0	1.0	1.0	1.0	1.0	8.0	47	51	0	0	107.5	112.5	FSIQ WISC-III or WAIS-III

© 2019 Rødgaard E-M et al. *JAMA Psychiatry.*

Nordahl	2013	1.0	1	0.0	0.0	1.0	1.0	0.0	1.0	5.0	121	50	0	1	NA	NA	Mullen Developpmental Quotient (DQ)
Nur Say	2014	0.0	1.0	1.0	1.0	2.0	1.0	1.0	1.0	8.0	15	15	0	1	102.3	100.6	FSIQ WISC-R or WAIS-R
Bolton	1994	1.0	1.0	[redacted]	[redacted]	[redacted]	[redacted]	[redacted]	[redacted]	2.0	27	[redacted]	1	0	NA	[redacted]	WAIS-R, WISC-R, Memll-Palmer, BPVS, Raven's = approximately 1/3 3049;1/3 50-69; 1/3 >70
Bailey	1995	0.5	NA	[redacted]	[redacted]	[redacted]	[redacted]	[redacted]	[redacted]	0.5	21	[redacted]	1	0	NA	[redacted]	Wechsler scales, Raven's Matrices. the Reynell scales, the British Picture Vocabulary Scale, and the Merrill-Palmer test
Woodhouse	1996	0.5	NA	[redacted]	[redacted]	[redacted]	[redacted]	[redacted]	[redacted]	0.5	37	[redacted]	0	0	NA	[redacted]	Non-verbal IQ, 4 groups of IQ = <IQ 35, IQ between 35-49, IQ between 50-70, >IQ 70
Davidovitch	1996	0.0	NA	[redacted]	[redacted]	[redacted]	[redacted]	[redacted]	[redacted]	0.0	148	[redacted]	1	1	NA	[redacted]	Developmental age based on Stanford-Binet Intelligence Scale or Bayley Scales of Infant Development

© 2019 Rødgaard E-M et al. *JAMA Psychiatry.*

Stevenson	1997	0.5	NA	[redacted]	0.5	100	[redacted]	1	0	NA	[redacted]	NA
Lainhart	1997	1.0	1.0	[redacted]	2.0	91	[redacted]	1	1	NA	[redacted]	The subjects were divided into four groups based on their nonverbal IQ: less than 30. 30 to 49. 50 to 69. and ~70.
Skjeldal	1998	0.0	NA	[redacted]	0.0	25	[redacted]	1	0	NA	[redacted]	Only present proportions of individuals with differents types of mental retardation
Fombonne	1999	0.5	NA	[redacted]	0.5	126	[redacted]	1	0	NA	[redacted]	Griffiths test = quotient obtained by dividing the developmental age by the chronological age / proportions of individuals with differents types of mental retardation
Ghaziuddin	1999	0.0	NA	[redacted]	0.0	20	[redacted]	0	0	NA	[redacted]	Only evaluate the presence of intellectual disability (IQ < 70)

© 2019 Rødgaard E-M et al. *JAMA Psychiatry.*

Fidler	2000	0.5	NA	[redacted]	0.5	41	[redacted]	1	1	NA	[redacted]	Vocabulary and Block Design subtests of the WISC-R or WAIS-R (group are equivalent, but they don't give score of each group
Miles	2000	0.5	1.0	[redacted]	1.5	137	[redacted]	1	1	64.8	[redacted]	Leiter, WISC-R, Stanford
Gillberg (a)	2002	1.0	0.0	[redacted]	1.0	50	[redacted]	1	1	69.0	[redacted]	WISC-R
Gillberg (b)	2002	1.0	0.0	[redacted]	1.0	50	[redacted]	0	1	97.0	[redacted]	WISC-R
Deutsch & Joseph	2003	0.5	1.0	[redacted]	1.5	63	[redacted]	1	1	76.0	[redacted]	DAS
Fuller Torrey	2004	0.0	0.0	[redacted]	0.0	15	[redacted]	1	0	56.9	[redacted]	NA
Dementieva	2005	0.5	NA	[redacted]	0.5	251	[redacted]	1	1	NA	[redacted]	NA
Lainhart	2006	0.5	1.0	[redacted]	1.5	338	[redacted]	0	1	102.8	[redacted]	Non-verbal IQ, WISC-III or WAIS-III, DAS, Leiter, Mullen
Sacco	2007	0.5	NA	[redacted]	0.5	241	[redacted]	1	1	NA	[redacted]	Raven, Bayley, Leiter
Van Daalen	2007	1.0	0.0	[redacted]	1.0	53	[redacted]	0	1	NA	[redacted]	Mullen = proportions of individuals with <70IQ, entre 70-84IQ et >85IQ only
Webb	2007	1.0	NA	[redacted]	1.0	28	[redacted]	0	1	NA	[redacted]	NA
Miles	2008	1.0	NA	[redacted]	1.0	172	[redacted]	1	1	NA	[redacted]	NA
Davidovitch	2011	0.0	NA	[redacted]	0.0	317	[redacted]	0	1	NA	[redacted]	NA
Chawarska	2011	1.0	1.0	[redacted]	2.0	98	[redacted]	0	1	82.2	[redacted]	Non-verbal Mullen
Ververi	2012	0.0	NA	[redacted]	0.0	222	[redacted]	1	0	NA	[redacted]	NA

© 2019 Rødgaard E-M et al. *JAMA Psychiatry.*

Froehlich	2013	0.5	NA	[redacted]	0.5	255	[redacted]	0	1.0	NA	[redacted]	Stanford-Binet 5th (only correlation between FSIQ and HC measure)
Chaste	2013	0.5	NA	[redacted]	0.5	1889	[redacted]	0	1	NA	[redacted]	Differential Ability Scales 2nd, WISC-IV, Mullen Scales of Early Learning, or the Raven's standard
Grandgeorge	2013	0.5	NA	[redacted]	0.5	422	[redacted]	0	1.0	NA	[redacted]	NA
Cederlund	2014	1.0	1.0	[redacted]	2.0	33	[redacted]	0	0	79.2	[redacted]	Developmental Quotient (DQ) only = Griffiths' Developmental scales

Since macrocephaly was compared to population-wide norms rather than individual control-groups, some NOS items could not be rated for the macrocephaly studies.

© 2019 Rødgaard E-M et al. *JAMA Psychiatry.*

eTable 19. NOS Rating Criteria

The Newcastle-Ottawa Scale (NOS) for Assessing the Quality of Studies Included - AUTISM adaptation			
Selection (Tot = 4)			
**Item 1:** Is the autism characterization adequate?			
.5 point with one standardised asessment (ADOS and/or ADI and/or CARS) in a majority of autistic participants (80% and over)			
.5 additional point if with standardized asessment + clinical judgment. "Clinical judgment" can be clinical interview, best estimate diagnosis, team of professionals.			
(Note: Diagnosis can be made previously or reconfirmed for the study. Also, a study with no standardised assessment gets a score of 0)			
**Item 2:** Representativeness of the cases			
1 point if IQ range of 40 or over among autistic participants or if SD (standard deviation) of IQ of 12 and over in autistic group (on at least one IQ measure available)			
**Item 3**: Selection of controls			
Attempt was made to recruit groups similar in terms of IQ and age (attempt at matching, no matter if in the end there is a group difference on one of these variables)			
**Item 4:** Definition of controls			
1 point if controls have no history of autism previously diagnosed. Ex: The participant section mentions that a questionnaire/interview asked about neurological conditions and/or psychiatric conditions and/or developmental conditions. Or the exclusion criteria mention autism and/or neurological/psychiatric/developmental conditions.			
If no description (not sure that autism has been formally excluded in the control group): 0 point			
Comparability (Tot = 2)			
**Item 5:** Comparability of cases and controls on the basis of the design or analysis			
1 point if matched on intelligence (any test) or developemental level (ex: Mullen, Bayley). (Note: if matched on the IQ of interest for the authors, give 1 point, even if there is another IQ measure with a between-group difference. Ex: groups matched on Performance IQ, but difference on Verbal IQ)			
.5 point if matched on gender/sex.			
.5 point if matched on chronological age.			
*matched means no significant between-group difference on the variable (if statistical test not reported, group means should be obviously close, within 0.25 SD)			
Outcome (Tot=3)			
**If more than 1 task/experiment in the study, or if many outcome measures (ex: score + response time + rating by examiner), please refer to the meta-analysis to verify which task was included in the meta-analysis.			
**Item 6:** Ascertainment of outcome			
1 point if Outcome is an objective measure (ex: score on a task, reaction time, volume of brain			

© 2019 Rødgaard E-M et al. *JAMA Psychiatry.*

structures) OR outcome rated by an evaluator who is blind to group. (note: most studies will get 1)			
outcome rated by an evaluator who is not blind to group = 0			
**Item 7:** Same task/procedure in both groups			
1 point if same task/procedure in both groups for the task/variable of interest (ex: theory of mind task, brain volume…)			
(Note: most studies will get 1. Example of 0 would be a study in which autistics get the full task/battery, and controls get a short version)			
**Item 8:** Loss of participants			
1 point if Loss of participants (ex: did not complete the task, technical difficulty, excluded for too low performance) is similar in both groups. "similar loss of participants" is when there is a maximum of 10% of between-group difference in loss of participants.			
(Note: only consider participants lost because they did not complete task or because their data could not be used. Do NOT consider participants excluded because they did not meet inclusion criteria. Ex: a participant that was recruited but that in the end had a too low IQ for inclusion)			
n autistic group			
number of participants used in analyses (after participant loss)			
if more than one autistic group, refer to meta-analysis to verify which group they kept or both			
n control group			
number of participants used in analyses (after participant loss)			
Autism group composition			
**Proportion of "strict" autism**			
Score 1 if majority of autistic participants have an "autism" (or High-functioning autism) diagnosis (minimum of 80% of the sample)			
Score 0 if ASD (autism spectrum disorder/condition), or Asperger, or PDD (Pervasive developmental disorder), or a mix of diagnoses			
**Exclusion of syndromic autism**			
Score 1 if it is mentioned that autistic participants with a known genetic condition, or neurological conditions, were excluded. If it is not mentioned, then it's 0.			
(Note: syndromic autism means with an identified genetic/neurologic condition like Fragile X or under identified mutations, or Tuberous sclerosis, etc. However, it will rarely be mentioned explicitely "syndromic autism" in the papers.)			
IQ			
**IQ autistic group:** mean IQ in autistic group (if more than one autistic group, refer to meta-analysis to verify which group they kept or both). Order of priority (take the first measure available in this order of priority): Full-Scale IQ, Performance IQ (Non-Verbal IQ), Verbal IQ, specific subtest of other measure of IQ (the one that is used for matching participants, ex: Raven's Matrices, Peabody Picture Vocabulary Test).			
**IQ control group:** Mean IQ control group (use same test as for autistic group)			
**IQ test:** subscale (if applicable: FSIQ, PIQ, VIQ, etc) and test (WAIS, WISC, WASI, WPPSI, RPM, DAS, etc.)			
FSIQ= Full-Scale IQ			
PIQ=Performance IQ or NVIQ=Non-Verbal IQ			
VIQ=Verbal IQ			

Studies using the same task were always scored by the same rater.

© 2019 Rødgaard E-M et al. *JAMA Psychiatry.*

eTable 20. Results of Analysis of Control Variables

The table shows the p-values (F-tests) for each control variable in each of the constructs. Numbers in parentheses show the p-value of publication year after adding the respective control variables.

	Brain size	Emotion Recognition	Cognitive Flexibility	Inhibition	ERP P3b	Planning	ToM
**Comparability score**	0.153 (0.004)	0.986 (0.008)	0.801 (0.159)	0.099 (0.817)	0.784 (0.025)	0.124 (0.028)	0.817 (0.0)
**IQ difference**	0.722 (0.004)	0.167 (0.007)	0.007 (0.127)	0.001 (0.804)	0.616 (0.024)	0.807 (0.036)	0.367 (0.0)
**Quality score**	0.06 (0.003)	0.384 (0.007)	0.404 (0.156)	0.185 (0.818)	0.51 (0.023)	0.327 (0.033)	0.399 (0.0)
**Strict autism**	0.755 (0.004)	0.68 (0.008)	0.979 (0.159)	0.504 (0.82)	0.459 (0.022)	0.404 (0.034)	0.812 (0.0)

© 2019 Rødgaard E-M et al. *JAMA Psychiatry.*

eTable 21. Quality of Meta-analyses—Social Domain

Inclusion and exclusion criteria of original studies in selected meta-analyses on social domain (emotion recognition and theory of mind constructs).

Constructs	Emotion recognition / Theory of mind	Emotion recognition / Theory of mind	Emotion recognition	Emotion recognition
References	**Chung 2014**	**Leppanen 2018**	**Penuelas-Calvo 2018**	**Uljarevic & Hamilton 2013**
Period of inclusion	Up to December 2011	From 1992 to 2017	Beginning with the inception of each database through February 28, 2017	Up to December 2011
Inclusion criteria				
Language of publications	Peer-reviewed journals in English	English	No restriction	Studies published in English
Age	Between 18 and 65 years	Adults or adolescents aged 12 years or older	No limitation	No limitation
Diagnoses	Mentalizing abilities in adults with diagnoses of schizophrenia, schizoaffective disorder or first-episode psychosis (SCZ) or ASD according to Research Diagnostic Criteria, DSM-III-R, DSM-IV or the International Classification of Diseases criteria	Diagnosis of ASD, Asperger’s Disorder (AS), high functioning autism (HFA), or AN	ASD diagnosis must be either confirmed by a clinician prior to participation in the study, using objective criteria acceptable at the time of publication or by using a standardized diagnostic tool	Participants formally diagnosed with Autistic Spectrum Condition
Controls	Healthy subjects as a comparison group	Age-matched healthy comparison (HC) group	IQ-matched controls	A group of typically developed subjects
Outcomes	Mentalizing tasks should be originally developed in autism and adopted to SCZ literature and used at least in 5 independent studies either for SCZ or for ASD	Assess theory of mind (refering to the ability infer information about others’ emotions, intentions, knowledge, and beliefs from social interaction or given information)<br>Studies that required the identification of complex emotions or emotional mental states, such as frustration, were included	A version of the “Reading the Mind in the Eye” test must be used	Studies examining recognition of emotions presented in the visual modality. Information regarding the accuracy on behavioural tasks had to be available in order for study to be included / Studies examining more than one of the six standard emotions (fear, surprise, anger, disgust, happiness and surprise expressed by face and body)
Other	Studies should report means and standard deviations, or F or t values, or exact P value so that standardized mean differences could be calculated	Studies should have at least ten participants in each group.	An IQ test or a similar intelligence test must be carried out	-
Exclusion criteria				
Type of publication	Not specified	Conference abstracts	Master’s and doctoral theses and conference presentations	Master and doctoral theses and conference presentations
Outcomes	-	Studies using tasks in which theory of mind ability was inferred from eye movements or reaction times / Studies that used self- report questionnaires or parental report measures to assess theory of mind / Studies that used tasks that produced error rates, such as the Penny Hiding Game / Studies that assessed theory of mind during functional magnetic resonance imaging (fMRI) or positron emission tomography / Studies that only investigated recognition of basic emotions	-	Complex or social emotions and recognition of emotional hand gestures
Other	Studies that report measures with dichotomous outcomes	Studies in which only young children took part	Intervention studies that could affect the RMET performance (i.e., training studies, Neurofeedback, etc.) / Studies with other psychiatric pathology associated either in controls or patients.	-

© 2019 Rødgaard E-M et al. *JAMA Psychiatry.*

eTable 22. Quality of Meta-analyses – Executive Domain

Inclusion and exclusion criteria of original studies in selected meta-analyses on executive domain (planning, inhibition and flexibility constructs).

Constructs	Planning	Planning / Inhibition / Flexibility	Flexibility	Flexibility	Inhibition
References	**Olde Dubbelink 2017**	**Lai 2016**	**Landry 2016**	**Westwood 2016**	**Geurts 2014**
Period of inclusion	Up to November 2015	From 1978 to 31 December 2015	Prior to February 2013	Up to and including January 2016	Before June 2013
Inclusion criteria					
Language of publications	Studies written in English and published in a peer-reviewed journal	Studies reported in English*	Not specified	English full-text	Studies published in a peer-reviewed journal and written in English.
Age	Not specified	Participants were children and adolescents (younger than or equal to 18 years of age)	Children or adults	No limitation	No limitation
Diagnoses	ASD participants were the population being studied and they met diagnostic criteria according to the DSM-III, DSM-III-R, DSM-IV, DSM-IV-TR, DSM-5, or ICD-10 (defined by clinical diagnosis, autism questionnaires, interviews or observation schedules)	ASD groups were diagnosed according to the International Classification of Disease (the 9th, 10th version) or Diagnostic and Statistical Manual of Mental Disorders (III, III-R, IV, IV-TR, V),	At least one participant group diagnosed with autism spectrum disorder*	Clinical population of either ASD or AN (from Diagnostic and Statistical Manual of Mental Disorders, 5th edition (DSM-V; American Psychiatric Association, 2013, with all variants of the two disorders included.	ASD participants had to meet diagnostic criteria according to the DSM-III-R, DSM- IV, or ICD-10 (defined by clinical diagnoses, autism questionnaires, interviews, or observation schedules)
Controls	A typically developing (TD) comparison group	Comparison group comprised healthy individuals	Not specified	Healthy controls	Typically developing (TD) control group
Outcomes	Experimental or clinical neuropsychological planning tasks	The neuropsychological assessments were carried out using valid and reliable tests with reference to Lezak [2012] and Strauss, Sherman, and Spreen [2006].	Experiments containing the Wisconsin Card Sort Test (WCST)*	Studies using the WCST and reporting the number or percentage of perseverative errors (PE)	Widely known experimental or neuropsychological inhibition tasks (inhibition, inhibitory control, interference, cognitive control, emotion, Stop task, Go/No-Go task, Stroop task, Simon task, Flanker task).
Other	Studies provided outcome data sufficient and suitable for the calculation of effect sizes, either in the published study or upon request<br>Articles presented original data	Sufficient information for computation of effect sizes, namely the mean, standard deviation, the number of subjects, or the t value<br>Primary case-control studies	Not specified	Not specified	Not specified
Exclusion criteria					
Type of publication	Not specified	Unpublished studies	Literature review studies or studies that did not provide any data of their own	Reviews	Not specified
Outcomes	Not specified	Use of questionnaires to quantify the deficits	Used modified version of the WCST used six cards instead of 10 for each rule category	Adapted WCST	Not specified
Other	Not specified	ASD participants that were not all high-functioning (IQ < 70)	Studies that did not provide the necessary information	Standardised, not raw scores reported	Studies that reported insufficient information to calculate the effect size

* these items were originally reported as exclusion criteria (or reasons for exclusion in the flow chart), but are transforming into inclusion criteria in this table for comparison between studies purposes.

© 2019 Rødgaard E-M et al. *JAMA Psychiatry.*

eTable 23. Quality of Meta-analyses—Neurological Domain

Inclusion and exclusion criteria of original studies in selected meta-analyses on neurological domain (P3b amplitude and brain size).

Constructs	P3b amplitude	Brain size (brain circumference)	Brain size (brain volume - MRI)	
References	**Cui 2017**	**Sacco 2015**	**Sacco 2015**	
Period of inclusion	Before 1st Sept 2015	Up to November, 2014	Up to November, 2014	
Inclusion criteria				
Language of publications	Article written in English, and full text published	Articles in English, German, French, Italian, or Spanish*	Articles in English, German, French, Italian, or Spanish*	
Age	No limitation	No limitation	No limitation	
Diagnoses	Autism spectrum disorder (ASD) participants	Assessing patients with idiopathic autism (i.e., DSM-IV diagnoses of either Autistic Disorder, Asperger's Disorder or Pervasive Developmental Disorder Not Otherwise Specified, PDD-NOS)	Assessing patients with idiopathic autism (i.e., DSM-IV diagnoses of either Autistic Disorder, Asperger's Disorder or Pervasive Developmental Disorder Not Otherwise Specified, PDD-NOS)	
Controls	Typically developed (TD) control group	Not specified	"Controls"	
Outcomes	Studies that used event-related potential technique to measure P300 components	Measuring head circumference in autistic patients and providing the percentage of macrocephalic individuals	Studies reporting structural MRI data, specifically TBV expressed as cc or ml where means and standard deviations were available or could be obtained	
Other	Both mean and standard deviation of the P300 component amplitude and/or latency of both ASD and TD groups had to be available directly, or calculable from other data forms in the content, tables or graphs	Not specified	Not specified	
Exclusion criteria				
Type of publication	Not specified	Case reports, commentaries and reviews<br>Retrospective or longitudinal studies of head circumference trajectory providing multiple data points per each individual	Case reports, commentaries and reviews<br>Studies providing longitudinal data of total brain volume	
Outcomes	Not specified	Publications lacking measures of head circumference, including clinical, neurocognitive, biochemical, brain imaging and post-mortem studies	Publications lacking measures total brain volume, including clinical, neurocognitive, biochemical, brain imaging and post-mortem studies<br>Publications reporting only volumetric data for specific or isolated brain regions or limited to gray or white matter. When both total brain volume or area were provided, only the former was considered<br>Studies reporting intracranial volume (ICV) and not total brain volume (TBV), whereby ICV also includes cerebrospinal fluid (CSF);<br>Studies employing other electrophysiological or neuroimaging techniques, including Diffusion Tensor Imaging, functional magnetic resonance imaging, proton magnetic resonance spectroscopy, Voxel Based Morphometry, Positron Emission Tomography, Single Photon Emission Tomography, and EEG brain mapping, or providing physical or neuroanatomical parameters other than TBV, including cortical thickness and cortical surface	
Other	ASD and/or TD groups that included participants with brain lesion or other mental disorders<br>ASD and/or TD groups that included participants being treated with antipsychotic medications or other relevant treatments at the period of ERP test<br>Sample size of each group less than 4	Studies on animal models or studies using a genetic approach;<br>Reports on known syndromic forms of autism, Rett syndrome or specific diagnoses other than idiopathic ASD<br>Reporting head circumference measurements of healthy individuals only	Studies on animal models or studies using a genetic approach;<br>Reports on syndromic autism, Rett syndrome or specific diagnoses other than idiopathic ASD ;<br>Reporting data only from from healthy individuals of from patients only ;<br>Reporting data of identical or overlapping previously-published data sets ;<br>Reporting data not provided by the authors as mean +/- SD	

* these items were originally reported as exclusion criteria (or reasons for exclusion in the flow chart), but are transforming into inclusion criteria in this table for comparison between studies purposes.

© 2019 Rødgaard E-M et al. *JAMA Psychiatry.*

eTable 24. Comparison of Meta-analysis Quality

Main differences between selection criteria of different meta-analyses, interpretation and risk of bias induced by the combination of data from several meta-analyses

Construct	Number of 1-to-1 comparisons	Main differences between selection criteria of different meta-analyses	Interpretation	Risk of bias induced by the combination of data from several meta-analyses
Theory of mind	1	The main difference is that Leppanen et al. included adolescents (aged 12 and older) **and** adults, while Chung et al. restricted the inclusion to studies conducted among adults **only**.	Not likely to be an issue, because combining data from both meta-analyses yields to a better representativeness of the autistic population (i.e. adolescents and adults)	Low, because the inclusion periods described by respective search strategies overlap significantly (see Table 21)
Emotion recognition	6	- Leppanen et al. included adolescents (aged 12 and older) **and** adults, Chung et al. restricted the inclusion to studies conducted among adults **only**. The two other meta-analyses did not limit the inclusion of studies based on age criteria.<br><br>- Leppanen et al allowed the inclusion of studies that required the identification of complex emotions, while Uljarevic et al. excluded such studies.	- Not likely to be an issue, because combining data from these meta-analyses yields to a better representativeness of the autistic population (i.e. children, adolescents and adults).<br><br>- Not likely to be an issue, because combining data from these meta-analyses yields to a broader assessment of the construct.	Low, because the inclusion periods described by respective search strategies overlap significantly (see Table 21)
Planning	1	No major differences between selection criteria	-	Low, because the inclusion periods described by respective search strategies overlap significantly (see Table 22)
Inhibition	1	No major differences between selection criteria	-	Low, because the inclusion periods described by respective search strategies overlap significantly (see Table 22)
Flexibility	3	No major differences between selection criteria	-	Low, because the inclusion periods described by respective search strategies overlap significantly (see Table 22)
P3b amplitude	0	As data were extracted from only one meta-analysis for this construct, there was no risk of differences between selection criteria from several meta-analyses.	-	N/A
Brain size	0	As we used data from only one meta-analysis for this construct, there was no risk of differences between selection criteria from several meta-analyses.	-	N/A

© 2019 Rødgaard E-M et al. *JAMA Psychiatry.*

eTable 25. Meta-analyses: Databases and Search Strategies

Databases and search strategies in meta-analyses

	Chung et al.	Cui et al.	Geurts et al.	Lai et al.	Landry et al.	Leppanen et al.	Westwood et al.	Olde Dubbelink et al.	Penuelas-Calvo et al.	Sacco et al.	Uljarevic et al
Databases	Pubmed, Medline, EMBASE, Sciencedirect	PubMed, Embase, Cochrane Library	PubMed/Medline, PsycINFO	MEDLINE, Embase, PsycINFO, Web of Science	PubMed	Pubmed, Scopus, Web of Knowledge, and OVID (PsycINFO, PsycARTICLES, MEDLINE, AGRIS, Embase)	PubMed, PsycINFO, Scopus, Web of Science	PubMed, PsycINFO, Web of Science	PubMed, PsycINFO, Clinicaltrials.gov	PubMed, Scopus, Google Scholar	PubMed, PsycINFO, Web of Science
Search strategy	'theory of mind', 'mentalising' or 'mentalizing', 'social cognition', 'faux pas', 'eyes test', 'strange stories', mindreading', 'reading the mind in the eyes' appeared with either 'autism*', 'autism spectrum disorders', 'schizophrenia' or 'psychosis' and their combinations	"((("Autistic Disorder"[Mesh]) OR "Child Development Disorders, Pervasive"[Mesh]) OR "Asperger Syndrome"[Mesh]) AND "Evoked Potentials"[Mesh]" + "(P300 OR P3a OR P3b OR P3) AND (autism OR autistic OR ASD OR Asperger)"	(autism, autistic disorder, pervasive developmental disorder, asperger, PDD-NOS, ASD), combined with (inhibition, inhibitory control, interference, cognitive control, emotion, Stop task, Go/No-Go task, Stroop task, Simon task, Flanker task)	"Autis*", "Asperger*", "Pervasive Development*", "ASD" or "PDD" were paired up with "Executive function*", "Inhibit*", "Cognitive control", "Interference control", "Working memory", "Visual memory", "Verbal memory", "Visuospatial memory", "Spatial memory", "Flexibility", "Shifting", "Planning", "Generativity" or "Fluency"	'Autism'' AND ''executive function'' OR ''card sort''	("autism spectrum disorder") OR "Asperger syndrome") AND ("theory of mind" OR mentalizing OR "Reading the mind in the eyes" OR "reading the mind in the voice" OR "reading the mind in the video").	anorexia nervosa OR autism AND set-shifting, Wisconsin, executive function OR cognitive flexibility	(autism; autistic disorder; pervasive developmental disorder; Asperger; ASD; PDD-NOS) combined with terms related to planning (planning; executive function; Tower; Tower of London (ToL); Tower of Hanoi (ToH); Stockings of Cambridge (SoC); Behavioral Assessment of the Dysexecutive Syn- drome (BADS); Mazes; CANTAB; WISC; NEPSY; D-KEFS; BRIEF)	("autism" OR "Asperger syndrome" OR "high functioning autism" OR "autism spectrum disorder" OR "pervasive developmen- tal" OR ASD) AND ("intelligence" OR "cognitive func- tion" OR cognition) AND ("emotional intelligence" OR "eye test" OR "reading the mind in the eyes" OR RMET OR "eye task" OR "theory of mind" OR "emotion recogni- tion" OR "facial expression" OR "facial affect" OR face OR eyes)	(autism OR autistic OR pervasive developmental disorders OR asperger) AND (head circumference OR cranial circumference OR macrocephaly OR head size OR megalencephaly) + (autism OR autistic disorder OR pervasive developmental disorders OR asperger) AND (volumetric magnetic resonance imaging OR brain volume).	combinations of the following terms: autism, Asperger syndrome, pervasive developmental disorders, emotion recognition, emotion perception, facial expression, facial affect, face, body.

© 2019 Rødgaard E-M et al. *JAMA Psychiatry.*

eTable 26. Reproducibility and Quality of the Search Strategies in Meta-analyses

Reproducibility and quality of the search strategies in meta-analyses

	Chung et al.	Cui et al.	Geurts et al.	Lai et al.	Landry et al.	Leppanen et al.	Westwood et al.	Olde Dubbelink et al.	Penuelas-Calvo et al.	Sacco et al.	Uljarevic et al
Reproducibility of the search strategy											
Named database provider	Y	Y	Y	Y	Y	Y	Y	Y	Y	Y	Y
Provided time period	N	N	N	Y	N	N	N	Y	Y	N	N
Specific year given for first date searched	*n*	*n*	*n*	*y*	*n*	*n*	*n*	*y*	*y*	*n*	*n*
Specific date given for last date searched	*y*	*y*	*y*	*y*	*y*	*n*	*y*	*y*	*y*	*y*	*y*
Indicated if limits were used	Y	Y	Y	Y	N	Y	Y	Y	Y	Y	Y
Limited to English articles	*y*	*y*	*y*	*y*	*n*	*y*	*y*	*y*	*n*	*n*	*y*
Limited to English and other language(s)	*n*	*n*	*n*	*n*	*n*	*n*	*n*	*n*	*y*	*y*	*n*
Limited to a certain type of publication	*n*	*n*	*n*	*n*	*n*	*n*	*n*	*y*	*y*	*n*	*n*
Provided specific search terms (complete search strategy with Boolean operators)	N	Y	N	Y	Y	Y	Y	N	Y	Y	N
Reproducibility assessment (number of "Y", maximum = 4)	2	3	2	4	2	3	3	3	4	3	2
Other standards of quality of the search strategy											
Attempts made at collecting unpublished data	N	N	N	N	Y	N	N	Y	N	N	N
Manual search conducted through references of articles, abstracts	N	N	N	Y	Y	Y	Y	Y	Y	Y	Y
Reporting/Conduct Standard Mentioned (e.g. PRISMA, MOOSE, MARS etc)	N	N	N	N	N	Y	Y	Y	Y	N	N
Flowchart included	Y	N	Y	Y	N	Y	Y	Y	Y	Y	Y
Indication of who conducted the search	N	Y	Y	Y	N	Y	Y	N	Y	N	N
Overall quality assessment (maximum=9)	3	4	4	7	4	7	7	7	8	5	4

Y: yes ; N: no

© 2019 Rødgaard E-M et al. *JAMA Psychiatry.*

eTable 27. Assessment of Publication Bias in Meta-analyses

Assessment of publication bias in included meta-analyses

Reference	Methods used to assess publication bias	Original results of the publication bias assessment for each meta-analysis (original quotations whenever possible)
Emotion recognition		
Chung	Funnel plot<br>Egger's test<br>Fail-safe analysis	"there might be some publication bias […] however, the fail-safe number of missing studies needed to make the group difference nonsignificant was large"
Leppanen	Begg's test	"significant publication bias was present"
Penuelas-Calvo	Funnel plot<br>Fail-safe analysis	"We did not find any significant evidence of publication bias"
Uljarevic	Funnel plot	"The clear asymmetry in the funnel plot […] suggests that publication bias may be an issue"
Theory of mind		
Chung	Funnel plot<br>Egger's test<br>Fail-safe analysis	"no publication bias"
Leppanen	Begg's test	"significant publication bias was present"
Cognitive flexibility		
Landry & Al Taie	Funnel plot<br>Fail-safe analysis	"Funnel plots suggest the potential for publication bias on [*1 on 4 variables*], whereas the funnel shape for the other outcome measures suggests the literature is representative
Lai	Funnel plot<br>Trim and fill method<br>Fail-safe analysis	Possibility of a publication bias for this construct, although hypothetical missing negative studies required to nullify the findings was regarded as acceptable.
Westwood	Funnel plot<br>Egger's test<br>Trim and fill method	« no evidence of publication bias »
Planning		
Olde	Funnel plot<br>Regression test<br>Trim and fill method<br>Fail-safe analysis	"we must consider a moderate impact of publication bias"
Lai	Funnel plot<br>Trim and fill method<br>Fail-safe analysis	No publication bias for this construct
Inhibition		
Geurts	Funnel plot<br>Regression test<br>Trim and fill method	"There is no evidently strong publication bias".
Lai	Funnel plot<br>Trim and fill method<br>Fail-safe analysis	No evidence of publication bias for this construct
P3b		
Cui	Funnel plot<br>Egger's test<br>Begg's test	"there is no publication bias »
Brain size		
Sacco	Egger's test	"Egger's regression test indicated no publication bias for this meta-analysis […] p-value=0.19"

© 2019 Rødgaard E-M et al. *JAMA Psychiatry.*

eFigure 1. Aggregate Publication Bias per Construct

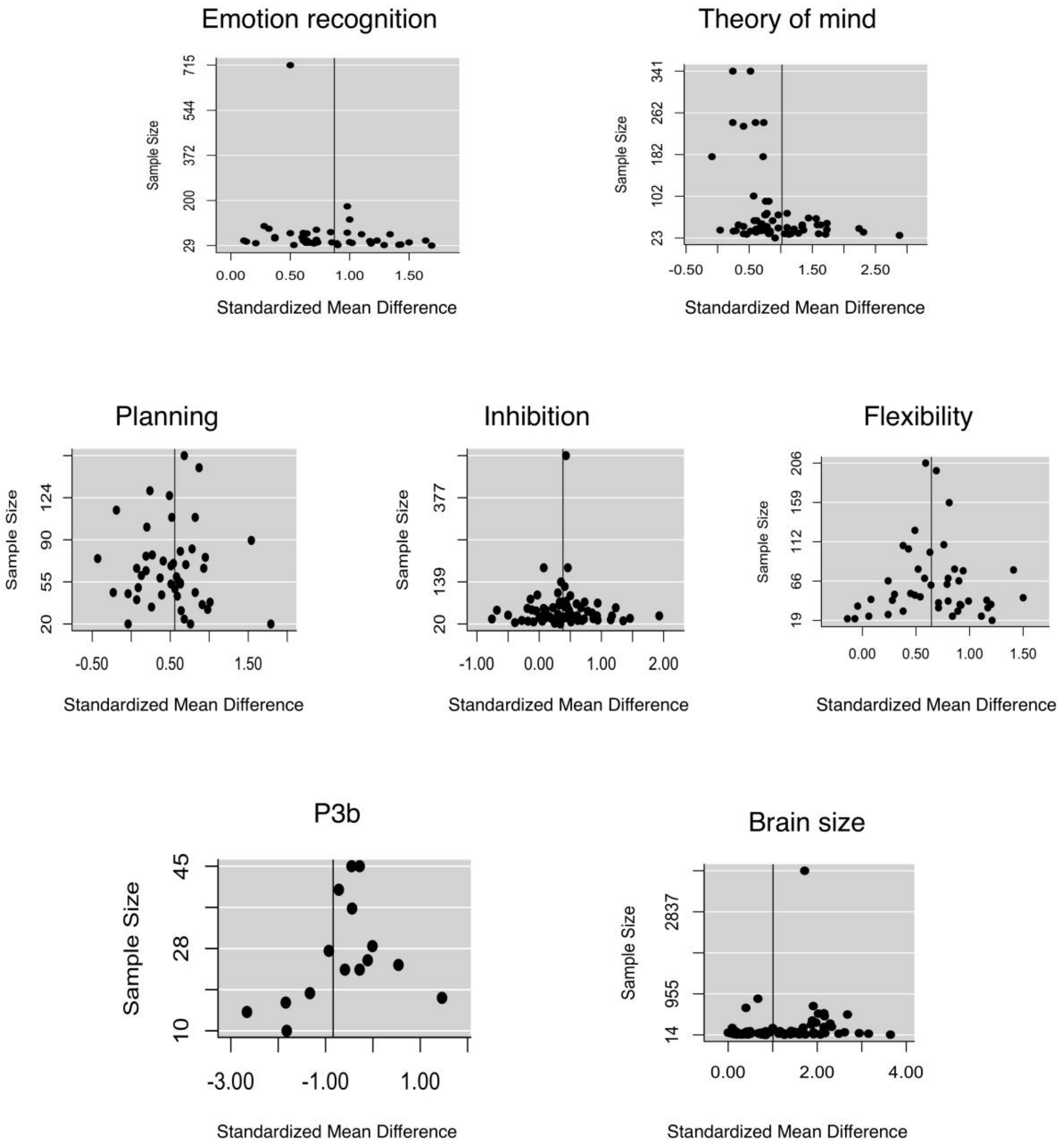

© 2019 Rødgaard E-M et al. *JAMA Psychiatry.*

eResults. Supplementary Results

Social domain

Data for the analysis of emotion recognition was obtained from meta-analyses conducted by Chung et al., Leppanen et al., Peñuelas-Calvo, and Uljarevic & Hamilton. From these meta-analyses, we analysed 64 effect sizes from studies published from 1989 to 2017, based on a total of 3,895 participants. A regression analysis, with task, sample size, and publication year as independent factors, resulted in a significant effect of publication year (see Table 1). The slope estimate of the temporal trend was -0.028, meaning that the effect size decreased over time.

Data for the analysis of theory of mind was obtained from studies conducted by Chung et al. and Leppanen et al.. We identified 62 effect sizes from studies published from 1992 to 2017, based on a total of 4,478 participants. For theory of mind, the temporal trend was significant, and the slope was estimated to be -0.045. For one task (strange stories), there was evidence of the Proteus phenomenon, as the first study, which found a much larger effect size than the other studies, had a studentized residual above the 95th percentile. We tested the influence of this data point by also performing the analysis without this study, which still showed a significant effect of publication year ($p < 0.001$), with a slope of -0.032.

Executive domain

We explored the three executive constructs cognitive flexibility, planning, and inhibition. The data on cognitive flexibility was obtained from three meta-studies conducted by Landry & Al-Taie, Lai et al., and Westwood et al.. We included 51 effect sizes from studies published from 1985 to 2015, based on a total of 3,137 participants. The slope for publication year was estimated to be -0.013. Effect sizes from one study, Ozonoff 1994 study 2, deviated substantially from those of almost all other studies and could thus be considered to be outliers. This unusual result was also noted by the authors themselves and a reproduction of the study (Ozonoff 1994, study 3) found the results to be consistent with the remaining literature. If the abnormal effect sizes were excluded from the analysis, the results changed markedly, with the slope being estimated to be -0.018, and the effect of publication year becoming significant ($p = 0.02$).

We examined the planning construct using data from meta-analyses of Olde Dubbelink & Geurts and Lai et al.. We included 46 effect sizes published from 1994 to 2015, based on a total of 3,033 participants. In addition to task type, the studies were sorted based on the applied outcome metric, as this varied between studies. The analysis of planning resulted in a significant slope for publication year of -0.067.

The construct inhibition was explored by analyzing data obtained from Geurts et al. and Lai et al.. We included 71 effect sizes from studies published from 1994 to 2015, based on a total of 4,460 participants. As with the analysis of planning, the studies were sorted by task and outcome metric. The slope for inhibition was estimated to be -0.003.

Neurological domain

Data for the analysis of P3b amplitude was obtained from a meta-analysis conducted by Cui et al.34. We included 14 effect sizes from studies published from 1980 to 2014, based on a total of 374 participants. The studies were partitioned by task type based on which modality was investigated within each study. The analysis of P3b amplitude resulted in a significant slope of -0.048.

Data for the brain size construct was obtained from a meta-analysis by Sacco et al.35. In total, 89 effect sizes were obtained from studies published from 1994 to 2014, based on a total of 8,326 participants. The brain size construct showed a significant decrease in effect size over time, with a slope of -0.047.

© 2019 Rødgaard E-M et al. *JAMA Psychiatry.*
